# Pathological high intraocular pressure induces glial cell reactive proliferation contributing to neuroinflammation of the blood-retinal barrier via the NOX2/ET-1 axis-controlled ERK1/2 pathway

**DOI:** 10.1186/s12974-024-03075-x

**Published:** 2024-04-22

**Authors:** Xin Shi, Panpan Li, Marc Herb, Hanhan Liu, Maoren Wang, Xiaosha Wang, Yuan Feng, Tim van Beers, Ning Xia, Huige Li, Verena Prokosch

**Affiliations:** 1 Department of Ophthalmology, Faculty of Medicine, University Hospital of Cologne, University of Cologne, 50937 Cologne, Germany; 2 Institute for Medical Microbiology, Immunology and Hygiene, Faculty of Medicine, University Hospital of Cologne, University of Cologne, Goldenfelsstr. 19-21, 50935 Cologne, Germany; 3 Cologne Cluster of Excellence on Cellular Stress Responses in Aging-Associated Diseases (CECAD), Cologne, Germany; 4 Department of Ophthalmology, Beijing Chaoyang Hospital, Capital Medical University, Beijing, 100020 P. R. China; 5 Institut I für Anatomie, Universitätsklinikum Köln (AöR), Cologne, Germany; 6 Department of Pharmacology, University Medical Center, Johannes Gutenberg University Mainz, Langenbeckstr. 1, 55131 Mainz, Germany; 7 German Center for Cardiovascular Research (DZHK), Partner Site Rhine-Main, 55131 Mainz, Germany

**Keywords:** NADPH oxidase 2, Deletion, Pharmacological inhibition, Oxidative stress, Neurodegeneration, Neuroinflammation, Vascular dysfunction, Glaucoma

## Abstract

**Background:**

NADPH oxidase (NOX), a primary source of endothelial reactive oxygen species (ROS), is considered a key event in disrupting the integrity of the blood-retinal barrier. Abnormalities in neurovascular-coupled immune signaling herald the loss of ganglion cells in glaucoma. Persistent microglia-driven inflammation and cellular innate immune system dysregulation often lead to deteriorating retinal degeneration. However, the crosstalk between NOX and the retinal immune environment remains unresolved. Here, we investigate the interaction between oxidative stress and neuroinflammation in glaucoma by genetic defects of NOX2 or its regulation via gp91ds-tat.

**Methods:**

Ex vivo cultures of retinal explants from wildtype C57BL/6J and *Nox2* ^*−/−*^ mice were subjected to normal and high hydrostatic pressure (Pressure 60 mmHg) for 24 h. In vivo, high intraocular pressure (H-IOP) was induced in C57BL/6J mice for two weeks. Both Pressure 60 mmHg retinas and H-IOP mice were treated with either gp91ds-tat (a NOX2-specific inhibitor). Proteomic analysis was performed on control, H-IOP, and treatment with gp91ds-tat retinas to identify differentially expressed proteins (DEPs). The study also evaluated various glaucoma phenotypes, including IOP, retinal ganglion cell (RGC) functionality, and optic nerve (ON) degeneration. The superoxide (O_2_^-^) levels assay, blood-retinal barrier degradation, gliosis, neuroinflammation, enzyme-linked immunosorbent assay (ELISA), western blotting, and quantitative PCR were performed in this study.

**Results:**

We found that NOX2-specific deletion or activity inhibition effectively attenuated retinal oxidative stress, immune dysregulation, the internal blood-retinal barrier (iBRB) injury, neurovascular unit (NVU) dysfunction, RGC loss, and ON axonal degeneration following H-IOP. Mechanistically, we unveiled for the first time that NOX2-dependent ROS-driven pro-inflammatory signaling, where NOX2/ROS induces endothelium-derived endothelin-1 (ET-1) overexpression, which activates the ERK1/2 signaling pathway and mediates the shift of microglia activation to a pro-inflammatory M1 phenotype, thereby triggering a neuroinflammatory outburst.

**Conclusions:**

Collectively, we demonstrate for the first time that NOX2 deletion or gp91ds-tat inhibition attenuates iBRB injury and NVU dysfunction to rescue glaucomatous RGC loss and ON axon degeneration, which is associated with inhibition of the ET-1/ERK1/2-transduced shift of microglial cell activation toward a pro-inflammatory M1 phenotype, highlighting NOX2 as a potential target for novel neuroprotective therapies in glaucoma management.

**Supplementary Information:**

The online version contains supplementary material available at 10.1186/s12974-024-03075-x.

## Background

Glaucoma, a heterogeneous group of disorders, is characterized by the loss of retinal ganglion cells (RGCs) and the axons, ultimately contributing to defects in the visual field. Glaucoma is considered the principal cause of irreversible blindness and is estimated to affect more than 111.8 million people by the year 2040 [1]. To date, the pathogenesis of glaucoma remains unclear, where multiple pathophysiological factors and pathways, which regulate mechanical, vascular, and immune responses, are considered critical events responsible for RGC apoptosis [2]. Besides, there is a potential pathological link between oxidative stress, endothelial function, and glaucoma [3]. Reactive oxygen species (ROS) function as vasodilators at low concentrations, while high concentrations may lead to vascular dysfunction [4]. Oxidative stress, induced by ROS overproduction, is associated with reduced ocular hemodynamics [5] and increased intraocular pressure (IOP) [6–8], possibly accounting for abnormal vascular permeability, which might explain the underlying mechanisms of these risk elements in the development of glaucoma. Ocular vascular dysregulation is another pathological feature of glaucoma, the underlying mechanism of which remains unclear. The phenomenon is probably attributed partially to autoregulation and endothelial dysfunction influenced by high IOP (H-IOP), which appears to be connected to oxidative stress [9].

The family of superoxide-generating enzymes, nicotinamide adenine dinucleotide phosphate (NADPH) oxidase (NOX), is considered to be a primary contributor to oxidative stress in various human pathologies [10]. NOX2, the primary NOX isoform of phagocytes, consists of membrane-bound (gp91^phox^, p22^phox^) and cytoplasmic (p40^phox^, p47^phox^, and p67^phox^) subunits [11, 12]. NADPH oxidase enzymes, act as an emerging source of oxidative stress in glaucoma, whose induction plays a key role in the progression of glaucoma [13]. Patients with glaucoma suffer compromised endothelial cells (ECs) function [14] and increased plasma and aqueous humor levels of endothelin-1 (ET-1) [15–17], which could lead to NOX activation [18]. Based on studies, under the circumstances of IOP, NOX2-dependent ROS production is associated with reduced endothelium-dependent relaxation of the retinal vasculature [19]. NOX2-derived “signaling ROS” are primarily involved in immune regulation and mediate protection against autoimmunity as well as maintenance of self-tolerance [20]. In the recent two decades, genetic NOX2 deletion has been investigated to clarify the impact of NOX2-derived ROS on disease development and progression. Pharmacological inhibition is also one of the methods commonly applied to investigate the contribution of NADPH oxidase, which has been extensively explored in animal models of vascular disorders [21]. However, in animal models of glaucoma, investigations of NOX2 are considerably limited [22], especially as the exact molecular mechanisms and potential effects on vascular endothelium and multiple downstream cytokines are unclear. Therefore, it is of high medical need to assess the effects of genetic deletion or pharmacological interventions with NOX2 in the animal models of glaucoma. The present study aimed to analyze the loss of RGC, whether it is associated with NOX2 activation in glaucoma, and whether suppressing or deleting NOX2 has a neuroprotective role in the animal model of glaucoma and the potential mechanisms.

## Materials and methods

### Laboratory animals

All animal experiments were conducted in accordance with the EU Animal Experimentation Directive 2010/63/EU and the Association for Research in Vision and Ophthalmology (ARVO) guidelines. In this study, the implemented animal protocol was scrutinized and approved by the government agency responsible for animal welfare in the state of North Rhine-Westphalia (Landesamt für Natur, Umwelt und Verbraucherschutz Nordrhein-Westfalen, Germany). The experiments were performed in C57BL/6J male mice (8–9 weeks old), *Nox2* ^*−/−*^ (*gp91* ^*phox−/−*^) and age-matched wildtype (WT) mice. The standard mouse housing conditions were: 12 h light/dark cycle, 22 ± 2 °C, 55 ± 10% humidity, and free access to food and water.

### In vivo episcleral vein occlusion model (H-IOP)

**Fig. 1:**
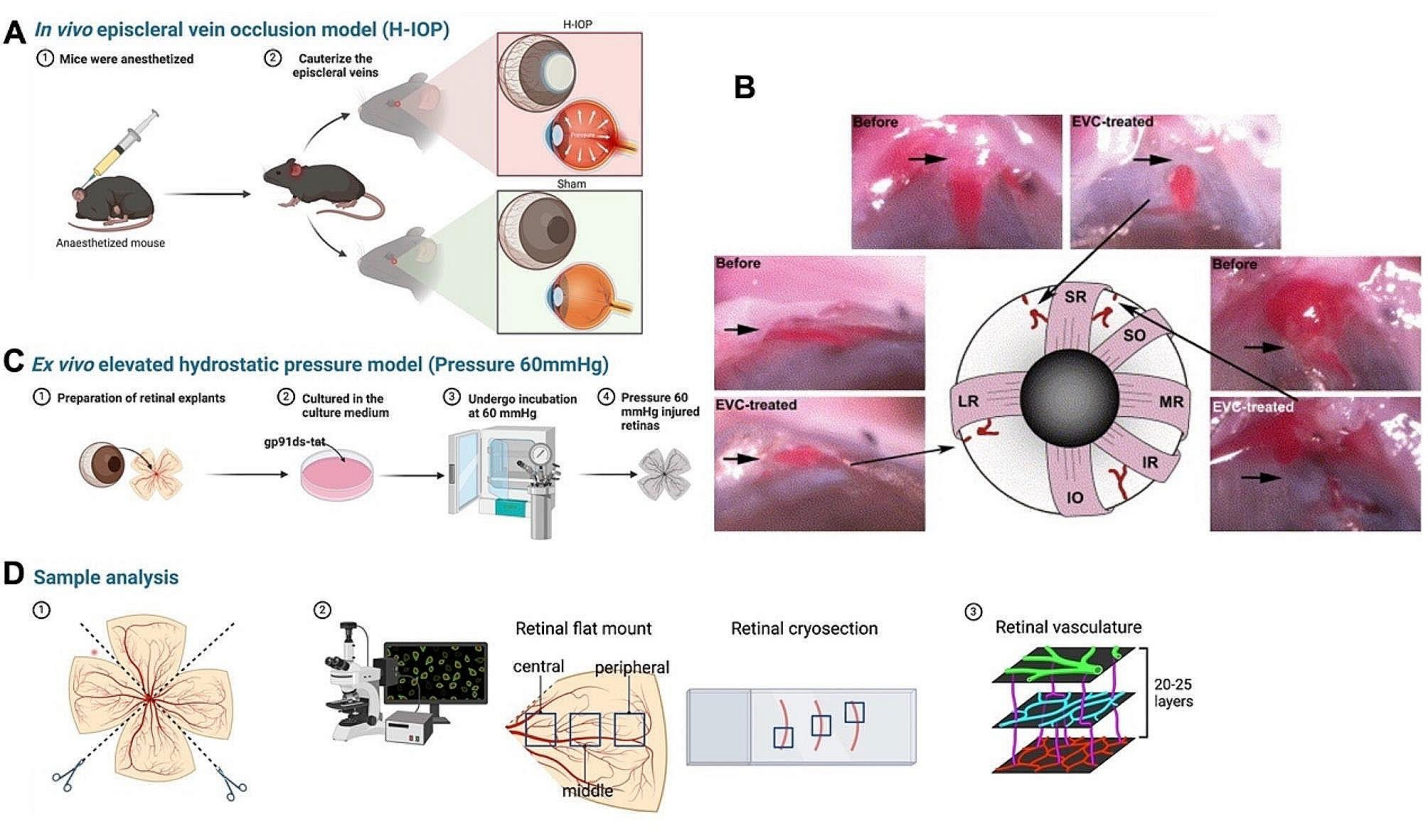
Schematic diagram of the construction of mouse glaucoma model and analysis of retinal samples. (**A**) The overview of in vivo H-IOP mouse model construction. (**B**) The schematic diagram of episcleral vein cautery. Image from Ruiz-Ederra et al., 2005 [23]. The illustration of the mouse eye depicts episcleral veins (red) relative to extraocular muscles, with three veins marked by cauterization interruptions. Pre- and post-cauterization (EVC-treated) images of these veins are provided, with arrows pointing to cauterization sites. (**C**) The overview of ex vivo retinal explants Pressure 60 mmHg model construction and inhibition of gp91ds-tat. (**D**) The overview of the retina sample preparation and the imaging and analysis of the retinal flat mount and cryosection. Abbreviations: EVC, episcleral vein occlusion; IO, inferior oblique; IR, inferior rectus; LR, lateral rectus; MR, medial rectus; SO, superior oblique; SR, superior rectus

All animals received episcleral vein occlusion surgeries in their right eyes (H-IOP) and sham surgeries in their left eyes (Sham) (Fig. 1A). The glaucoma mouse model was induced by occlusion of the three episcleral veins in their right eyes [19, 23]. Briefly, mice were anesthetized using a ketamine (100 mg/kg) and xylazine (10 mg/kg) solution via intraperitoneal injection. We also applied one drop of the 4 mg/mL oxybuprocain hydrochloride (Novesine® 0.4% Eyedrops, OmniVision®, OmniVision GmbH, Puchheim, Germany) onto the scathe ocular surface for local anesthesia. A cut was made across the conjunctiva and Tenon’s capsule at the limbal edge of the right eye under an operating microscope. Two relaxing incisions were made at the edges of the initial incisions, and the tissue was recessed posteriorly to expose the underlying extraocular superior and lateral rectus muscles. These muscles were gently pulled aside with a suture, bringing the episcleral veins into view. After surgical isolation, microforceps were positioned under the episcleral veins adjacent to the lateral and superior rectus and to the superior oblique muscles. A hand-held ophthalmic thermal cautery device (Fine Science Tools GmbH, Heidelberg, Germany) was employed to cauterize each vein until venous congestion was noticeable, indicating blockage without any leakage (Fig. 1B, the Image from *Ruiz-Ederra et al., 2005*). Great care was taken to minimize blood loss and avoid damage to the conjunctiva and the underlying sclera. Finally, the conjunctiva was put back to its original location, and ofloxacin ointment was given onto the ocular surface to prevent inflammation. Sham surgery was conducted similarly but without causing damage to the episcleral veins. Euthanasia via cervical dislocation was performed on the mice at three distinct post-operative intervals: 4 days, 8 days, and 2 weeks.

### IOP monitoring

The IOP of the mice was monitored using a TonoLab rebound tonometer (iCare, Vantaa, Finland) in animals once every two days [24, 25]. All measurements were conducted between 9:00 a.m. to 12:00 p.m. for comparability. After six consecutive measurements, the tonometer generated a mean IOP. We took 5 sets of measurements and averaged these IOP values.

### Ex vivo elevated hydrostatic pressure model (Pressure 60 mmHg)

The C57BL/6J, *Nox2* ^*−/−*^, and WT mice were euthanized by cervical dislocation. Eyes were immediately enucleated and transferred to Petri dishes containing ice-cold phosphate-buffered saline (PBS) (Carl Roth,1108.1), followed by peeling out the intact retina and dissecting the vitreous humor. The retinal explants were divided equally into four segments, ensuring that the ganglion cells were facing upward, and placed in Mixed Cellulose Ester (MCE) Membrane Filters (Advantec, A045R047Z-P). The retinal explants were transferred to lumox culture dishes 35 (Sarstedt, Nümbrecht, Germany). Retinal tissues were cultured in the standard culture medium, Dulbecco’s Modified Eagle’s Medium/Nutrient Mixture F-12 (DMEM/F12; Gibco BRL, Eggenstein, Germany) supplemented with 10 µg/mL porcine insulin, 100 U/mL penicillin, 100 µg/mL streptomycin. To minimize variability, retinal tissues were randomly assigned to the control and Pressure 60 mmHg groups.

For the Pressure 60 mmHg model, retinal explants undergo incubation for 8,16,24 h in a tailor-made pressure incubator chamber at a hydrostatic pressure of 60 mmHg, which simulates intraocular conditions under abnormally elevated IOP. Pressure incubation chambers with lids and non-directional valves allow access to 5% CO_2_ in the incubator at 37 °C for constant monitoring of internal air pressure employing manometers. Control retinas were cultured in the incubator with humidified 5% CO_2_ at 37 °C under normal atmospheric pressure (Fig. 1C).

### gp91ds-tat pharmacological intervention for experimental glaucoma models

The peptide gp91ds-tat (YGRKKRRQRRRCSTRIRRQL-NH2), a NOX2-specific inhibitor, interferes with NADPH oxidase assembly by targeting a sequence essential for binding gp91^phox^ with p47^phox^ [26, 27]. HIV-tat peptide, an amino acid sequence internalized by all cells, is linked to the gp91^phox^ sequence to facilitate cell entry [28]. For the Pressure 60 mmHg model, the retinal explants from C57BL/6J mice were incubated by adding different concentrations of gp91ds-tat (Anaspec, San Jose, CA, USA) in the standard culture medium for 24 h (Fig. 1C). Control or Pressure 60 mmHg retinas were incubated in the standard culture medium (Vehicle). For the in vivo H-IOP model, the different concentrations of gp91ds-tat (dissolved in normal saline) were injected intraperitoneally (10 ml/kg) 30 min after the H-IOP surgery and once every two days until the end of the experiment. Sham and H-IOP mice were injected with normal saline (Vehicle) (10 ml/kg). The mice were euthanized by cervical dislocation after two weeks. The preparation of retinal explants was performed as described above.

### Immunostaining of retinal flat mounts and cryosections

Each retina was divided into four equal sections along the dotted line in the schematic of Fig. 1D-1. Then, each quarter of the retinal explants from different mice served as an independent sample of each group, which was subjected to immunostaining for different markers and preparation of frozen sections. Retinal explants were fixed in 4% paraformaldehyde (PFA) (Histofix, Roth, Karlsruhe, Germany) for 30 min after rinsing with PBS. Subsequently, the retinas were rinsed twice with PBS for 10 min and dehydrated in 30% sucrose solution at 4 °C for 24 h to be analyzed further. After washing 3 times (10 min each) in PBS, the retinas were incubated in a buffer solution containing 1% BSA and 0.3% Triton-X-100 in PBS for 2 h at room temperature. Then, the retinas were incubated overnight at 4 °C with the primary antibody (Table 1). Next, the retinas were washed with PBS and placed with the secondary antibody in 1% BSA and 0.3% Triton-X-100 in PBS for 2 h at room temperature. Afterward, the retinas were rinsed with PBS three times and mounted on slides.

After fixation, the retinas were embedded in an optimal cutting temperature (OCT) compound (Sakura Finetek, Torrance, CA, USA) for cryostat sectioning. Retinal explants were cut vertically to a thickness of 12 μm by a Leica CM3050S cryostat (Leica Microsystems, Buffalo Grove, IL), collected onto gelatin-coated slides, and stored frozen until immunohistochemical processing. The retinal sections were washed with PBS for 5 min over 3 times. Then, the retina was blocked with 3% Normal goat serum and 0.1% Triton X-100 in PBS for 60 min, followed by primary antibodies (Table 1) at 4 °C for overnight incubation. After washing with PBS for 5 min 3 times, ensure that all procedures are performed in the dark and apply the secondary antibody 1:1000 in PBS for 2 h. Sections were rinsed with PBS. Finally, the retinal flat mounts and cryosection slides were mounted with VECTASHIELD® mounting medium with DAPI (BIOZOL Diagnostica Vertrieb GmbH, Eching, Germany) and covered with a coverslip.

**Table 1:** Antibodies used for histological analyses

Marker	Company	Dilution	Secondary antibody
Brn3a (mouse)	Millipore, MAB1585, Lot#3,684,607, EMD Millipore Corporation, Temecula, CA, USA	1:200	goat anti-mouse IgG H + L (Alexa Flour 488; ab150113, Abcam, Cambridge, UK)
Iba1 (rabbit)	019-19741, FUJIFILM Wako Pure Chemical Corporation	1:500	goat anti-rabbit IgG H + L (Alexa Flour 594; ab150080, Abcam, Cambridge, UK)
CD31 (mouse)	MEC13.3, BD	1:200	goat anti-mouse IgG H + L (Alexa Flour 488; ab150113, Abcam, Cambridge, UK)
NOX2 (rabbit)	ab129068, Abcam, Cambridge, UK	1:200	goat anti-rabbit IgG H + L (Alexa Flour 594; ab150080, Abcam, Cambridge, UK)
ET-1 (mouse)	NB300-526, Novus Biologicals, Littleton, USA	1:200	goat anti-mouse IgG H + L (Alexa Flour 488; ab150113, Abcam, Cambridge, UK)
GAFP (rabbit)	#Z0334, Agilent (DAKO)	1:200	donkey anti-rabbit IgG H + L (Alexa Flour 647; ab150075, Abcam, Cambridge, UK)
Isolectin GS-IB4	Alexa Fluor 488 conjugate, Invitrogen	1:500	-
α-SMA	ab202368 Abcam, Cambridge, UK Alexa Fluor® 594 Anti-alpha smooth muscle Actin antibody (1A4)	1:200	-
p-ERK1/2 (rabbit)	#9101, Cell Signaling	1:300	goat anti-rabbit IgG H + L (Alexa Flour 594; ab150080, Abcam, Cambridge, UK)

### Imaging and analysis of retinal flat mounts and cryosections

**Fig. 2:**
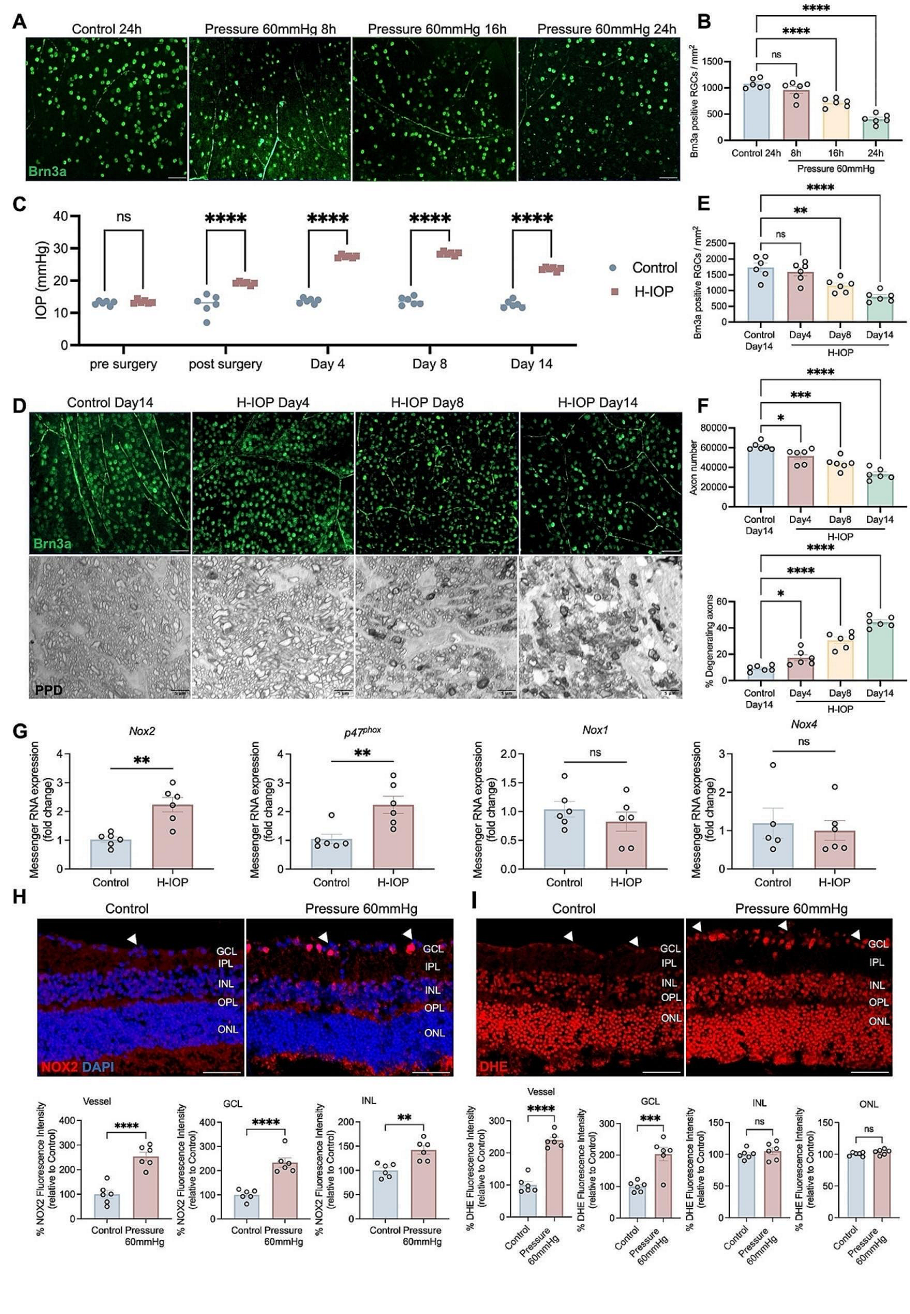
Pathologically high intraocular pressure-induced glaucomatous RGC loss and neurodegeneration. (**A**) Representative images of flat mount retina immunostained with Brn3a after Pressure 60 mmHg. Scale bar, 50 μm. (**B**) Analysis of the number of Brn3a-positive RGCs at different times after Pressure 60 mmHg. Data are shown as mean ± SEM (*n* = 6 in each group, one-way ANOVA with Tukey’s multiple comparisons test, *p < 0.05,**p < 0.01, ***p < 0.001, ****p < 0.0001). (**C**) Time course of IOP before as well as 14 days after H-IOP surgery. Data are shown as mean ± SEM (*n* = 6 in each group, two-way ANOVA with Šídák’s multiple comparisons test, ****p < 0.0001). (**D**) Representative images of flat mount retina immunostained with Brn3a and PPD-stained ON after H-IOP. Scale bar, 50 μm and 5 μm. (**E**) Analysis of the number of Brn3a-positive RGCs at different times after H-IOP. (**F**) Analysis of the number of axons and the percent of degenerating axons in the different groups. Data are shown as mean ± SEM (*n* = 6 in each group, one-way ANOVA with Tukey’s multiple comparisons test, *p < 0.05,**p < 0.01, ***p < 0.001, ****p < 0.0001). (**G**) Messenger RNA expression of Nox enzymes (Nox2, p47^phox^, Nox1, Nox4) in H-IOP retinas. Data are presented as the fold-change after H-IOP versus control. Data are shown as mean ± SEM (*n* = 6 in each group, Unpaired T-test, *p < 0.05,**p < 0.01, ***p < 0.001, ****p < 0.0001). (**H**) Representative images of retinal cross-sections immunostained with NOX2 after Pressure 60 mmHg (Scale bar, 50 μm) and the analysis of NOX2 fluorescence intensity in the retinal vessels and different retinal layers. (**I**) Representative images of retinal cross-sections immunostained with DHE staining after Pressure 60 mmHg. Scale bar, 50 μm. Analysis of DHE fluorescence intensity in the retinal vessels and different retinal layers. The white arrows point to cross-sections of retinal blood vessels. Data are presented as the percent fluorescence intensity of the Pressure 60 mmHg versus control. Data are shown as mean ± SEM (*n* = 6 in each group, Unpaired T-test, **p < 0.01, ****p < 0.0001)

Images were captured using a Zeiss Imager M.2 equipped with an Apotome.2 (Carl Zeiss; Jena, Germany). As detailed in Fig. 1D-2, three areas—central, middle, and peripheral—were imaged under a 20X magnification for each quarter of the retinal flat mount. Similarly, for retinal cryosections, the Zeiss Imager M.2 with an Apotome.2 was employed to photograph the central, middle, and peripheral regions of three consecutive sections from each sample, using a 20X objective.

**Fig. 3:**
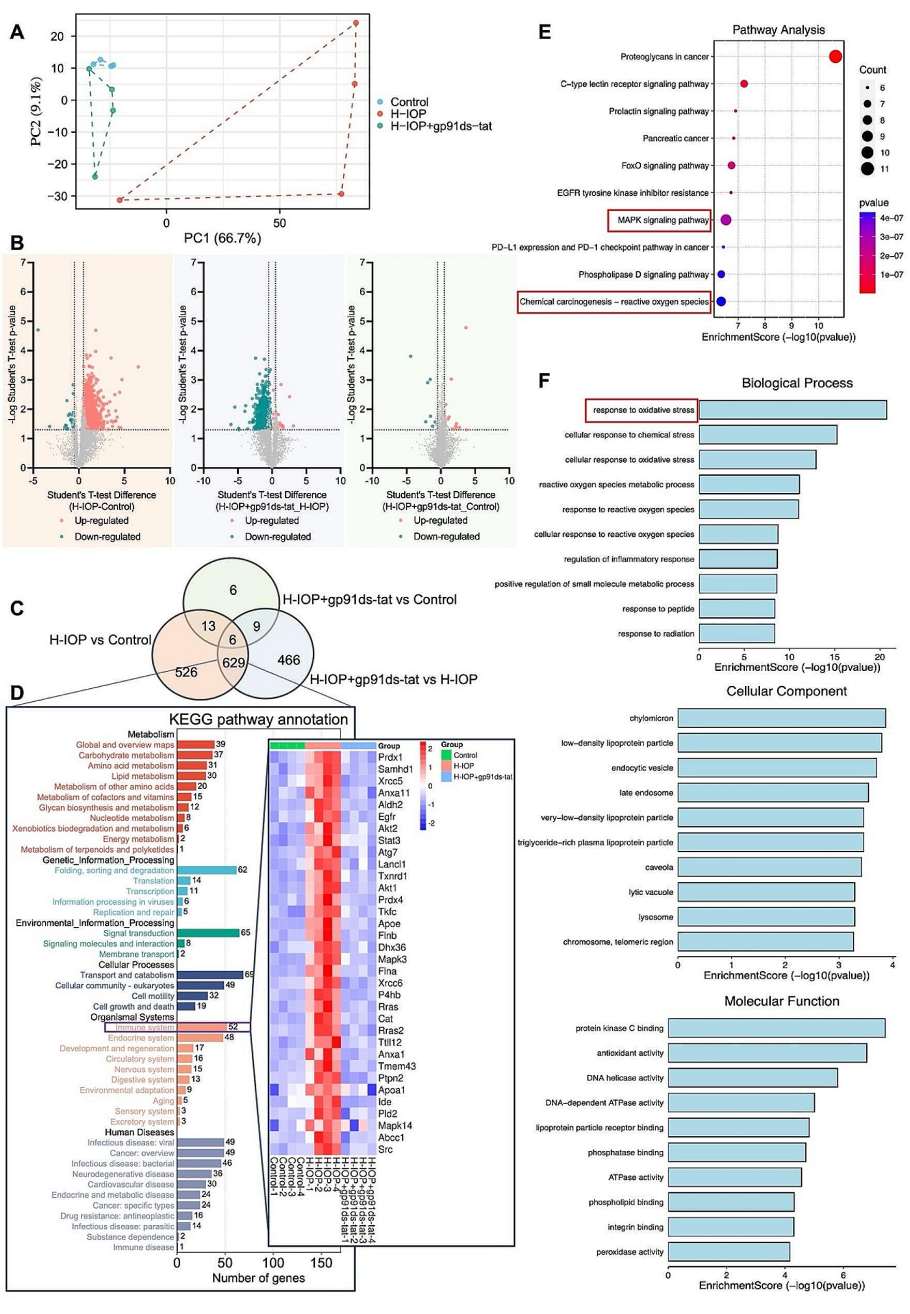
gp91ds-tat treatment effectively modulates the expression of major immune-related genes in H-IOP injured retina. (**A**) Principal component analysis shows the clustering of proteins in different samples. (**B**) Volcano plot showing differentially expressed proteins between H-IOP and control group, H-IOP + gp91ds-tat and H-IOP, H-IOP + gp91ds-tat and control group. (**C**) The Venn diagram was created by the differentially expressed data of proteins. (**D**) The most enriched KEGG Level 2 pathways for the 635 co-differentially expressed proteins. Hierarchical clustering illustrates distinct expression differences of immune genes between the three groups and homogeneity between groups. (**E**) The 10 most enriched KEGG pathways for the differentially expressed proteins. The size of the symbol represents the number of genes, and the colors represent the *p*-value. (**F**) The 10 most enriched gene ontology terms for the parental genes of the differentially expressed immune genes. Enriched GO terms are on the vertical axis, and the number of annotated differentially expressed genes associated with each GO term is indicated on the horizontal axis

Glia-vascular unit interactions in the retinal flat mounts were examined using a Leica SP8 confocal laser-scanning microscope (Leica, Wetzlar, Germany) at 20X magnification. Additionally, CD31 immunolabeled retinal flat mounts were observed with a Leica SP8 confocal microscope. Z-stack images, comprising 20–25 layers for each area (Fig. 1D-3), were compiled along the Z-axis to produce a 2D representation of the retinal vasculature.

Image analysis was conducted using ImageJ2 2.3.0 (http://rsb.info.nih.gov/ij/), NIH, Bethesda, MD, USA. Quantitative assessments of various markers, including RGCs count et al., were performed. The mean count from the three regions of a quarter-section of the retina provided a measure of overall retinal changes, which was then utilized for further statistical evaluation.

### Culture and stimulation of primary microglia

As described previously [29], primary microglia were isolated from C57BL/6J neonatal mouse pups (P0). Cell suspension was plated on cell culture dishes (Nunc) coated with Poly-D-Lysine (#A003E stock 1 mg/ml (use 50ng/ml in PBS)) in PM Medium (Neurobasal-A, 10% horse serum, B27, P/S/G). Primary Microglia at passage 2 or 3 were used for experiments. Cells were stimulated with 100nM ET-1 (E7764, Sigma-Aldrich) and inhibited with 20 μm MEK (PD98059) for 20 min and 24 h starting at 3 days in vitro (DIV). For Immunostaining, primary microglia grown on slides were treated and then washed with PBS, followed by fixation with 4% PFA for 30 min. The immunostaining protocol for primary microglia follows the same procedure as used for the retinal flat-mount.

### Quantification of cytosolic ROS

Retinas were also homogenized in lysis buffer as previously described [30]. Lysates were centrifuged at 1500 g for 3 min, and the supernatant was exposed to 20 µM 2,7-dichlorofluorescein derivative 6-carboxy-2,7-dichlorodihydrofluorescein diacetate, di(acetoxymethyl ester) (referred to as DCF throughout this manuscript) for 30 min at 37 °C. Fluorescence was measured using a plate reader (Tecan infinite 200Pro) (ex/em = 485/535 nm).

### Quantification of superoxide (O_2_^−^)

O_2_^-^ levels were measured in frozen sections of unfixed retinas by staining with the fluorescent dye dihydroethidium (DHE) [19, 31–33]. Immediately after thawing, tissue sections were incubated with 1 µM of DHE for 30 min at 37 °C. The slides were mounted with VectaShield mounting medium (Vector Laboratories, Burlingame, CA) and covered with a coverslip. Photographs of retinal cross-sections were taken using a Leica SP8 confocal laser scanning microscope (Leica, Wetzlar, Germany). As described previously, the staining intensity of blood vessels and individual retinal layers was measured using ImageJ software (NIH, http://rsb.info.nih.gov/ij/).

### Assessment of optic nerve degeneration

Optic nerve (ON) degeneration was assessed by p-phenylenediamine (PPD)-stained ON cross sections, as previously described [34, 35]. The semi-thin cross sections of ON were taken from 1.0 mm posterior to the eyeballs. In brief, the ON was separated from the eyeball and fixed overnight in a phosphate-buffered 3% glutaraldehyde/paraformaldehyde mixture at 4 °C. Following overnight treatment in 1% osmium tetroxide at 4 °C, ON were rinsed in 0.1 M phosphate buffer and 0.1 M sodium-acetate buffer, then dehydrated in graded ethanol concentrations. After embedding ON in resin (Eponate-12; Ted Pella), 1 μm sections were cut and stained in 1% PPD for 10 min. The sections were imaged under a Zeiss Axio Imager Z1 Microscope (Zeiss, Oberkochen, Germany) with a Zeiss Plan-ACHROMAT 100×Lens (Zeiss, Oberkochen, Germany). Image Acquisition was performed using a Canon EOS 6D Mk II camera (Canon, Krefeld, Germany) and CanonEOS Utility Software (Canon, Krefeld, Germany). Following a protocol similar to that of retinal cryosection imaging, three fields (including one central and two peripheral) were imaged from three sequential cross-sections for each ON, utilizing a 100× magnification. ON axon numbers were counted manually with ImageJ software (http://rsb.info.nih.gov/ij/, NIH, Bethesda, MD, USA), and the average numbers were calculated as axon density per square millimeter of ON and then multiplied by cross-sectional area to calculate the total number of axons per ON [31].

### Western blotting

Tissues were processed for Western blotting as previously described [36]. In brief, the retinal tissues and primary microglia were washed twice with cold PBS and incubated in 100 µL lysis buffer (T-PER Tissue Protein Extraction Reagent + 2% protease inhibitor) per whole retina explants on ice. After 15 min of incubation, the retina and cell lysates were dissected and sonicated for 1 min in an ultrasonic bath on ice and then centrifuged at 1000 g for 8 min. The supernatants were collected for further analysis. The BCA Protein Assay Kit (Pierce, Rockford, IL, USA) determined each lysate’s protein concentration per the manufacturer’s instructions. From each retina lysate, 20 µg of protein was loaded onto a Novex NuPAGE 12% Bis-Tris polyacrylamide gel (Thermo Fisher, USA). The gel electrophoresis was run using NuPAGE running buffer MES at room temperature with a voltage of 130 V for 60 min. After electrophoresis, the proteins were transferred to polyvinylidene fluoride membranes (Bio-Rad, Gladesville, Australia) using a dry transfer system (Bio-Rad, Hercules, CA, USA), and a standard transfer buffer (with 20% methanol), a voltage of 20 V was applied for 7 min. For immunoblotting, membranes were incubated with the appropriate antisera ET-1 (1:1000, NB300-526, Novus Biologicals, Littleton, USA), p47^phox^ (1:1000, sc-17,845, Santa Cruz Biotechnology), gp91^phox^ (1:1000, sc-130,543, Santa Cruz Biotechnology), polyclonal antibodies recognizing phospho-JNK (Thr183/185) (1:1000; catalog no. 9251), phospho-ERK1/2 (Thr202/204) (1:1000; catalog no. 9101) and phospho-p38 MAPK (Thr180/182) (1:1000; catalog no. 4511) overnight at 4 °C, and labeling was carried out using a multi-step detection procedure. First, appropriate biotinylated secondary antibodies were reacted with membranes, and then streptavidin-peroxidase conjugates were applied. Blots were developed with a 0.016% solution of 3-amino-9-ethyl carbazole in 50 mM sodium acetate (pH 5) containing 0.05% Tween-20 and 0.03% H_2_O_2_. Images were acquired from labeled blots and analyzed for densitometry using the software program ImageJ2 2.3.0. Densitometry values were then normalized for β-actin (1:10000, ab6276, Abcam).

### MS measurement

Proteins were extracted from the retinal tissue of these individuals using the detergent sodium dodecyl sulfate (SDS), which is the most efficient reagent for lysing tissue and cells to achieve complete protein extraction [37]. Subsequently, DIA-MS proteomics analysis was performed with a high-resolution, high-quality precision LTQ-Orbitrap Elite mass spectrometer. The continuum MS data were collected by an ESI-LTQ Orbitrap XL-MS system (Thermo Scientific, Bremen, Germany) and searched against the UniProt database with MaxQuant software version 1.5.3.30 (Max Planck Gesellschaft, Germany). Due to the random nature of “Birdshot” label-free quantitative proteomics, protein identification or abundance data are sometimes missing in some samples [38]. 40 A target-decoy-based false discovery rate (FDR) was set to 0.01 for the identification of peptides and proteins, the minimum peptide length was 6 amino acids, and the minimum unique peptides were set at 2. Fold changes of the label-free quantitation (LFQ) intensities were calculated to identify the significantly differentially expressed proteins (DEPs). We performed differential expression analysis on the quantitative proteomics data targeting the H-IOP relative to the control |Student’s T-test Difference| ≥ 0.5 change threshold and -Log *P*-value > 1.3. To outline the DEPs profiles of the transcripts, generating volcano maps via the R ggplot2 package and performing hierarchical cluster analysis via the Manhattan distance metric and the Ward minimum variance method from the heatmap package in R. Principal component analysis (PCA), implemented in the prcomp function of R, was conducted to abstract the main characteristics of the data, which served as an indicator of the overall state of the data.

### Gene ontology and kyoto encyclopedia of genes and genomes pathway analysis

Gene Ontology (GO; http://geneontology.org/) analysis is based on three annotated ontologies, including the exploration of molecular function (MF), cellular components (CC), and biological processes (BP), which originate from DEPs targeting genes. Simultaneously, the Kyoto Encyclopedia of Genes and Genomes (KEGG; http://www.kegg.jp/) analysis was performed to assess the biological functions and enrichment pathways of the DE genes. GO and KEGG analyses were performed by assigning R packages based on hypergeometric distributions.

### Enzyme-linked immunosorbent assay (ELISA)

The concentration of cytokines in total retinal and primary microglia lysates was measured by ELISA. Tissue samples were sonicated in PBS supplemented with protease and phosphatase inhibitors (Complete protease inhibitor cocktail, Roche). Interleukin 1beta (IL-1β) (DY401), tumor necrosis factor-alpha (TNF-α) (DY410), and interleukin 6 (IL-6) (DY406) Quantikine® ELISA′s were purchased from R&D Systems.

### Quantification of gene expression by quantitative PCR

Messenger RNA for the pro-oxidant enzymes, the NOX enzymes (*Nox1*, *Nox2*, *p47* ^*phox*^, *Nox4*), for the vascular endothelial growth factor-A (*Vegf-a*), for the antioxidant redox enzymes, heme oxygenase 1 (*Ho-1*), and glutathione peroxidases 1 (*Gpx1*), for the cytokines, *Tnf-α*, *IL-1β*, superoxide dismutase type 2 (*Sod2*), and for the nitric oxide synthase (NOS) isoforms, *eNos*, *iNos*, and *nNos*, was quantified in the whole retinal explants as described before [39]. Tissue samples were homogenized (FastPrep; MP Biomedicals, Illkirch, France). RNA was isolated using peqGOLD TriFast™ (PEQLAB), and cDNA was generated with the High-Capacity cDNA Reverse Transcription Kit (Applied Biosystems, Darmstadt, Germany). Quantitative real-time RT-PCR (qPCR) reactions were performed on a StepOnePlus™ Real-Time PCR System (Applied Biosystems) using SYBR® Green JumpStart™ Taq ReadyMix™ (Sigma-Aldrich, Munich, Germany) and 20 ng cDNA. Relative mRNA levels of target genes were quantified using the comparative threshold (CT) normalized to the housekeeping gene TATA-binding protein (*Tbp*). The qPCR primer sequences are shown in Table 2.

**Table 2:** Primer sequences used for quantitative PCR analysis

Gene	Accession number	Forward	Reverse
*Nox1*	NM_172203.1	GGAGGAATTAGGCAAAATGGATT	GCTGCATGACCAGCAATGTT
*Nox2*	NM_007807.2	CCAACTGGGATAACGAGTTCA	GAGAGTTTCAGCCAAGGCTTC
*p47* ^*phox*^	NM_001286037.1	AGAGCACGGATGGCACAAAG	CCGCGGGCTGTGGTT
*Nox4*	NM_015760.2	GGCTGGCCAACGAAGGGGTTAA	GAGGCTGCAGTTGAGGTTCAGGACA
*Ho-1*	NM_010442	GGTGATGCTGACAGAGGAACAC	TAGCAGGCCTCTGACGAAGTG
*Vegf-a*	NM_001025250.3	ACTTGTGTTGGGAGGAGGATGTC	AATGGGTTTGTCGTGTTTCTGG
*Gpx1*	NM_008160	CCCGTGCGCAGGTACAG	GGGACAGCAGGGTTTCTATGTC
*Sod2*	NM_013671	CCTGCTCTAATCAGGACCCATT	CGTGCTCCCACACGTCAAT
*Tnf-α*	NM_001278601.1	GCCTCTTCTCATTCCTGCTTG	CTGATGAGAGGGAGGCCATT
*Il-1β*	NM_008361	AAGGAGAACCAAGCAACGACAAAA	TGGGGAACTCTGCAGACTCAAACT
*eNos*	NM_008713	CCTTCCGCTACCAGCCAGA	CAGAGATCTTCACTGCATTGGCTA
*iNos*	NM_010927	CAGCTGGGCTGTACAAACCTT	CATTGGAAGTGAAGCGTTTCG
*nNos*	NM_008712	TCCACCTGCCTCGAAACC	TTGTCGCTGTTGCCAAAAAC
*Tbp*	NM_013684	CTTCGTGCAAGAAATGCTGAAT	CAGTTGTCCGTGGCTCTCTTATT

### Statistics

Details of the statistical test used for each experiment are in figure legends, along with n and *p*-value. All data is represented as mean ± SEM. Statistical analysis was performed using GraphPad Prism 9 (GraphPad Software, La Jolla, California, USA).

## Results

### H-IOP injury-induced glaucomatous RGC loss and neurodegeneration H-IOP injury provoked up-regulation of NOX2 expression accompanying O_2_^-^ over-production in the retina

To elucidate the effect of H-IOP on glaucoma, we first assessed the survival of the Brn3a-positive RGC by establishing a corresponding model ex vivo explant cultures (Pressure 60 mmHg) and in vivo (H-IOP). Compared with the control group (0 mmHg, 24 h), Pressure 60 mmHg induced retinal damage, resulting in a decrease in RGC survival from 16 h, peaking at 24 h with an RGC loss rate of 62.35% (*n* = 6, *p* < 0.0001) (Fig. 2A, B). In addition, we induced mice sustainability H-IOP by episcleral vein occlusion. IOP ascends to a maximum within six days post-H-IOP surgery with a mean value of 29.33 ± 0.20 mmHg (*p* < 0.0001), which indicates successful induction of H-IOP glaucoma. By Day 14, the mean IOP progressively dropped to a mean value of 23.60 ± 0.23 mmHg (*p* < 0.0001), which was still above the physiologic IOP value (13.43 ± 0.2275 mmHg) (Fig. 2C).

Correspondingly, RGCs declined from Day 8 and peaked at Day 14 (803 ± 66.20/mm^2^) compared to sham (1734 ± 141.7/mm^2^) (*n* = 6, *p* < 0.0001) (Fig. 2D, E). Furthermore, PPD staining of ON cross-sections indicated a reduction in the total number of myelinated axons induced by H-IOP injury from Day 4, scattered vacuole formation on Day 8, and a significant vesicle and glial scar formation on Day 14 (Fig. 2F). Interestingly, ON degeneration belongs to an early event in glaucoma that appears to pre-date the loss of RGC. In conclusion, these data confirm that the H-IOP will contribute to the loss of RGC and ON degeneration in a time-dependent manner. Inspired by the above results, Pressure 60 mmHg–24 h and H-IOP-14-day retinas were selected for follow-up experiments to ensure the reliability of the experimental glaucoma model.

Besides mitochondria, the NOX enzymes [40] have been identified as one of the major sources of oxidative stress in retinal eye diseases such as ischemic retinopathy and age-related macular degeneration [41]. Aiming to determine the potential role of NOX enzymes in H-IOP-induced glaucomatous damage, we analyzed the transcription levels of different *Nox* isoforms by qPCR. The results show that the transcription levels of *Nox2* and *p47* ^*phox*^ statistically increased in the retina after H-IOP injury (Fig. 2G). In contrast, the mRNA levels of *Nox1* or *Nox4* were not significantly altered. Consistent with H-IOP-induced retinal injury, in the ex vivo Pressure 60 mmHg model, we observed a robust increase in NOX2 immunoreactivity compared to the control, which was specifically localized to the retinal vessels, the ganglion cell layer (GCL) and the inner nuclear layer (INL) (Fig. 2H). Interestingly, DHE staining assays indicated that Pressure 60 mmHg damage strongly stimulated the overproduction of O_2_^−^, which highly overlapped with the strong fluorescence region of NOX2 (Fig. 2I). These data imply that H-IOP-induced retinal injury might be associated with NOX2-mediated oxidative stress.

### The gp91ds-tat treatment modulates oxidative stress-driven pro-inflammatory signaling in H-IOP-injured retinas

To fully characterize the proteomic profiles associated with experimental glaucoma in mice, we performed proteomic analysis on retinal samples from control mice, H-IOP mice, and treated with gp91ds-tat mice (*n* = 4). The PCA maps illustrated the overall reproducibility and individual heterogeneity of protein expression profiles between Control, H-IOP, and H-IOP retinas treated by gp91ds-tat (Fig. 3A). We identified 1174 DEPs in the H-IOP compared with Control, 1110 DEPs in the H-IOP with gp91ds-tat compared with H-IOP, and 34 DEPs in the H-IOP with gp91ds-tat compared with Control (Fig. 3B). As shown in Fig. 3C, by Venn analysis, we screened 635 co-DEPs between the data set of H-IOP compared with Control and H-IOP with gp91ds-tat compared with H-IOP. Subsequent KEGG secondary pathway enrichment analysis of the 635 DEPs revealed that the Immune system was the most highly enriched in the Organismal Systems category (Fig. 3D). NOX2-derived ROS can affect pro-inflammatory signaling [42]. As shown in Fig. 3D, the gp91ds-tat treatment effectively reduced the expression of most immune-related genes in H-IOP-injured retinas. KEGG enrichment analysis revealed the C-type lectin receptor signaling pathway, MAPK signaling pathway, and reactive oxygen species (Fig. 3E). Notably, GO enrichment analysis also revealed that experimental glaucoma involves multiple BP, CC, and MF, which intimately correlated with response to oxidative stress and inflammatory response (Fig. 3F).

### NOX2 deficiency or pharmacological inhibition via gp91ds-tat salvages H-IOP injury-induced glaucomatous RGC loss and neurodegeneration but not IOP modulation

**Fig. 4:**
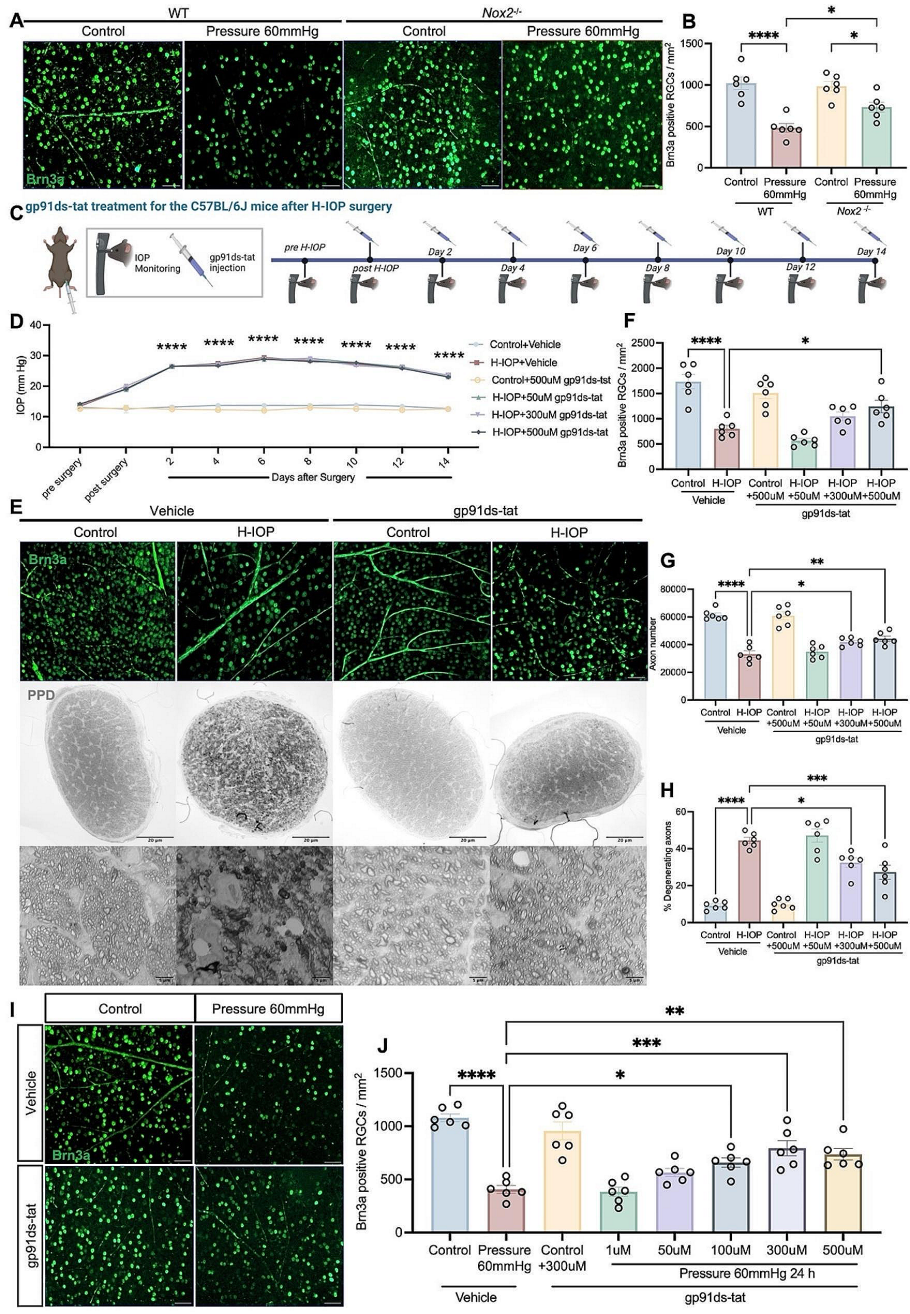
Pharmacological inhibition of NOX2 attenuates Pathologically high intraocular pressure-induced glaucomatous RGC loss and ON degeneration but not IOP modulation. (**A**) Representative images of WT and Nox2^−/−^ retinas immunostained with Brn3a after Pressure 60 mmHg 24 h. Scale bar, 50 μm. (**B**) Analysis of the number of Brn3a-positive RGCs in the different groups. Data are shown as mean ± SEM (*n* = 6 in each group, one-way ANOVA with Tukey’s multiple comparisons test, *p < 0.05, **p < 0.01,***p < 0.001, ****p < 0.0001). (**C**) The timeline of C57BL/6J mice after H-IOP with gp91ds-tat treatment and IOP measurement. (**D**) IOP measurements revealed sustained significant IOP elevation in H-IOP mice with gp91ds-tat-injected compared to Veh-injected control mice. Data are shown as mean ± SEM (*n* = 6 in each group, two-way ANOVA with Tukey’s multiple comparisons test, ****p < 0.0001). (**E**) Representative images of flat mount retina immunostained with Brn3a after H-IOP treated by 500µM gp91ds-tat. Scale bar, 50 μm. Representative images of PPD-stained ON after H-IOP treated by 500µM gp91ds-tat, at low (upper row, scale bar, 20 μm) and high (lower row, scale bar, 5 μm) magnification. (**F**) Analysis of the number of Brn3a-positive RGCs of retinas treated by different concentrations of gp91ds-tat after H-IOP in the different groups. (**G**) Analysis of the number of axons of ON treated by different concentrations of gp91ds-tat after H-IOP in the different groups. (**H**) Analysis of the percent of degenerating axons of ON treated by different concentrations of gp91ds-tat after H-IOP in the different groups. Data are shown as mean ± SEM (*n* = 6 in each group, one-way ANOVA with Tukey’s multiple comparisons test, *p < 0.05, **p < 0.01,***p < 0.001, ****p < 0.0001). (**I**) Representative images of flat mount retina immunostained with Brn3a after Pressure 60 mmHg treated by 300µM gp91ds-tat. Scale bar, 50 μm. (**J**) Analysis of the number of Brn3a-positive RGCs of retinas treated by different concentrations of gp91ds-tat after Pressure 60 mmHg in the different groups. Data are shown as mean ± SEM (*n* = 6 in each group, one-way ANOVA with Tukey’s multiple comparisons test, *p < 0.05, **p < 0.01,***p < 0.001, ****p < 0.0001)

To further explore the contribution of NOX2 in glaucoma, we initially tracked changes in RGC applying *Nox2* ^*−/−*^ mice. Compared with WT retinas, the survival of RGCs in *Nox2* ^*−/−*^ retinas improved by 52% after 24 h at Pressure 60 mmHg (Fig. 4A, B). Meanwhile, intraperitoneal injection of gp91ds-tat in mice significantly attenuated H-IOP-induced Brn3a-positive RGC loss in a dose-dependent manner but failed to modulate IOP (Fig. 4C-F). In addition, gp91ds-tat-treated effectively ameliorated H-IOP-induced ON degeneration, which included a 34% increase in the total number of axons (44,390 ± 1843 vs. 33,220 ± 2334, *n* = 6, *p* = 0.0058); and a 17% increase in the percentage of degenerated axons (27.33 ± 3.879% versus 44.50 ± 1.648%, *n* = 6, *p* = 0.0006) (Fig. 4E, G, H).

Finally, to support the knockout outcome by translational therapeutic approaches, we analyzed the RGC-protective effect of gp91ds-tat ex vivo. Treatment with 100µM, 300µM, and 500µM gp91ds-tat enhanced RGC survival by 62%, 95%, and 81%, respectively, compared to controls (*p* < 0.05) (Fig. 4I, J). Therefore, 300µM gp91ds-tat was considered the optimal therapeutic dose under in vitro conditions and was set as the standard treatment for subsequent experiments (*** *p* < 0.001). These data demonstrate that the absence of NOX2 and pharmacological inhibition effectively attenuates pathologically glaucomatous ON degeneration and reverses the loss of RGCs induced by H-IOP.

### NOX2 deficiency or inhibition dampens O_2_^-^ over-production induced by H-IOP in the vascular and GCL of the retina

**Fig. 5:**
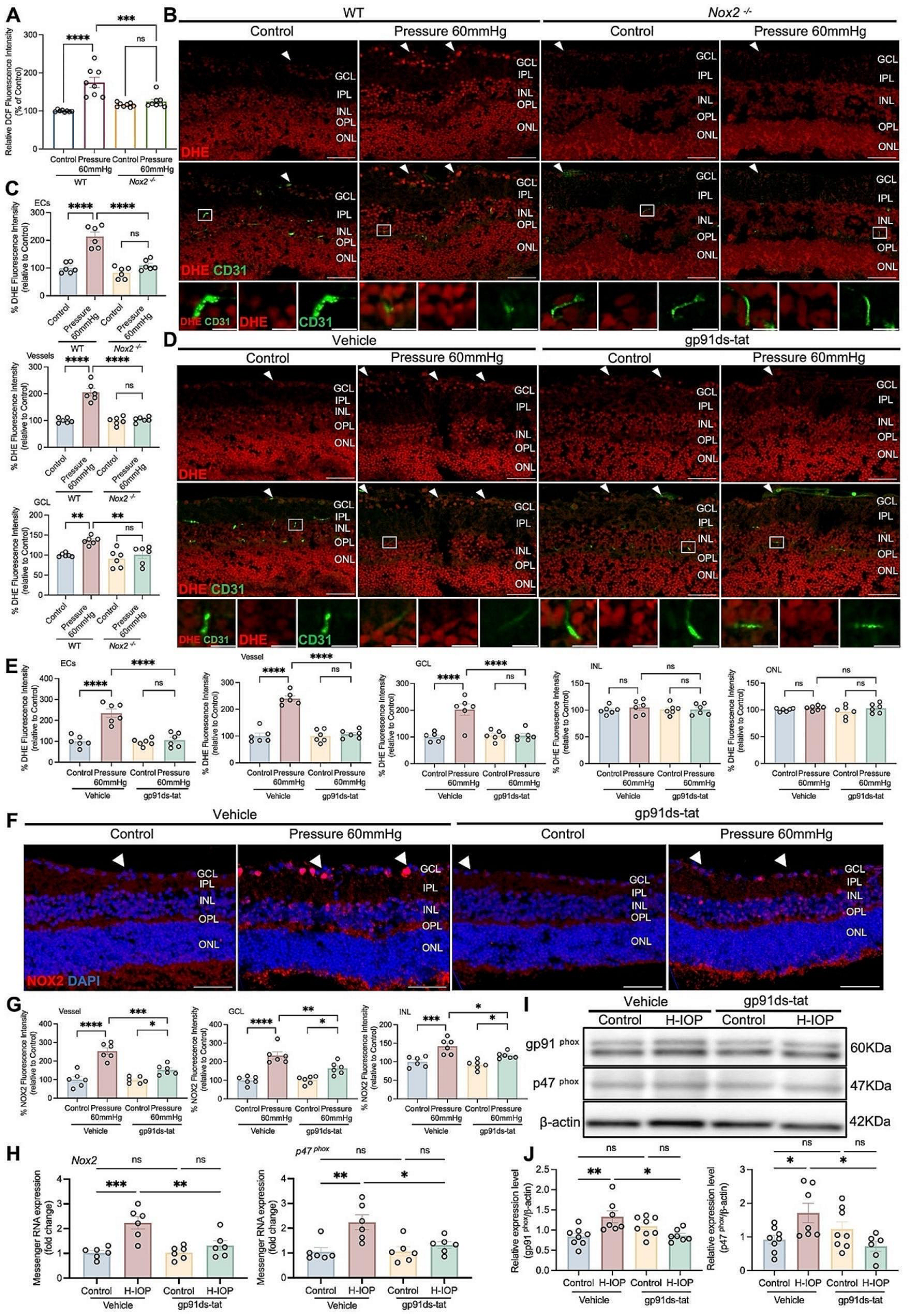
gp91ds-tat treatment down-regulates the NOX2 overexpression induced by Pressure 60 mmHg-injured in the retinal vessels and the GCL. (**A**) Retinal ROS production was measured using DCF diacetate probe. Data are presented as the percent fluorescence intensity of the groups versus the WT-Control. (**B**) Representative images of DHE and CD31 co-stained retinal cryosections from WT and Nox2^−/−^retinas after Pressure 60 mmHg 24 h. Scale bar, 50 μm. The white arrows point to cross-sections of retinal blood vessels. Lower panels in (**B**) show the enlarged views of the boxed regions (Scale bars equal 10 μm). (**C**) Analysis of DHE staining intensity in ECs, vessels, and GCL. Data are presented as the percent fluorescence intensity of the groups versus the control. (**D**) Representative images of DHE and CD31 co-stained retinal cryosections from Pressure 60 mmHg 24 h retinas with 300µM gp91ds-tat treatment. Scale bar, 50 μm. The white arrows point to cross-sections of retinal blood vessels. Lower panels in (**D**) show the enlarged views of the boxed regions (Scale bars equal 10 μm). (**E**) Analysis of DHE staining intensity in ECs, vessels, GCL, INL and ONL. Data are presented as the percent fluorescence intensity of the groups versus the control. (**F**) Representative images of retinal cross sections immunostained with NOX2 after Pressure 60 mmHg with 300µM gp91ds-tat treatment. The white arrows point to cross-sections of retinal blood vessels. Scale bar, 50 μm. (**G**) Analysis of NOX2 fluorescence intensity in the retinal vessels and GCL. Data are presented as the percent fluorescence intensity of the groups versus control-Vehicle. (**H**) Messenger RNA expression of Nox2 and p47^phox^in retinas after H-IOP and with 500µM gp91ds-tat treatment. Data are presented as the fold-change versus control-vehicle. (**I**) After H-IOP and with 500µM gp91ds-tat treatment, retinal extracts were assayed for Western blot analysis of gp91^phox^ and p47^phox^. Representative Western blots are presented. (**J**) The results obtained from Western blot are expressed as the ratio to β-actin from 6–9 independent experiments. Data are shown as mean ± SEM (*n* = 6 in each group, one-way ANOVA with Tukey’s multiple comparisons test, *p < 0.05, **p < 0.01,***p < 0.001, ****p < 0.0001)

NOX2-mediated ROS are an essential source of oxidative stress, whose excessive accumulation leads to retinal damage [43]. By quantifying the superoxide produced in the retina by reacting with DCF, we identified that Pressure 60 mmHg significantly evoked WT retinal superoxide production, which could be prevented by *Nox2* ^*−/−*^ (Fig. 5A). The results support the hypothesis that NOX2-mediated ROS is a key event in oxidative stress in the Pressure 60 mmHg retina.

To further clarify the primary location of NOX2-mediated ROS production in the H-IOP retina, we co-stained DHE with CD31 in the retinal cryosection to delineate oxidative stress derived from EC (Fig. 5B). *Nox2* ^*−/−*^ or treatment with gp91ds-tat significantly suppressed the overproduction of O_2_^-^ by EC, vasculature, and GCL at Pressure 60 mmHg (Fig. 5B-E). Notably, NOX2-mediated modulation of ROS was particularly prominent in the endothelium, where up to 2-fold excess O_2_^-^ was almost entirely inhibited by *Nox2* ^*−/−*^ or gp91ds-tat. In contrast, there are no significant changes in other layers (Fig. 5E, *Additional file 1*). The data show that pathologically H-IOP-induced NOX2-mediated ROS overproduction dominantly derives from ECs, blood vessels, and GCL.

Next, we attempted to elucidate the substantial pharmacologic role of gp91ds-tat repression of the NOX2 enzyme activity in glaucoma. The gp91ds-tat strongly attenuated the immunoreactivity augmentation by Pressure 60 mmHg injury-mediated NOX2, which was selectively localized to the vessel sites, GCL and INL by immunofluorescence staining in retinal cryosections (Fig. 5F, G). In addition, the transcript levels of *Nox2* and *p47* ^*phox*^ were significantly reduced in the H-IOP retinas treated with gp91ds-tat compared to the vehicle (Fig. 5H). Western blot analysis also supports that gp91ds-tat effectively reduces the expression levels of gp91^phox^ and p47^phox^ (Fig. 5I, J). These data indicated that gp91ds-tat does not only inhibit the activation of NOX2 but also reduces its protein expression.

### NOX2/ROS-activated glial cells involved in H-IOP-mediated blood-retinal barrier damage and inflammation

**Fig. 6:**
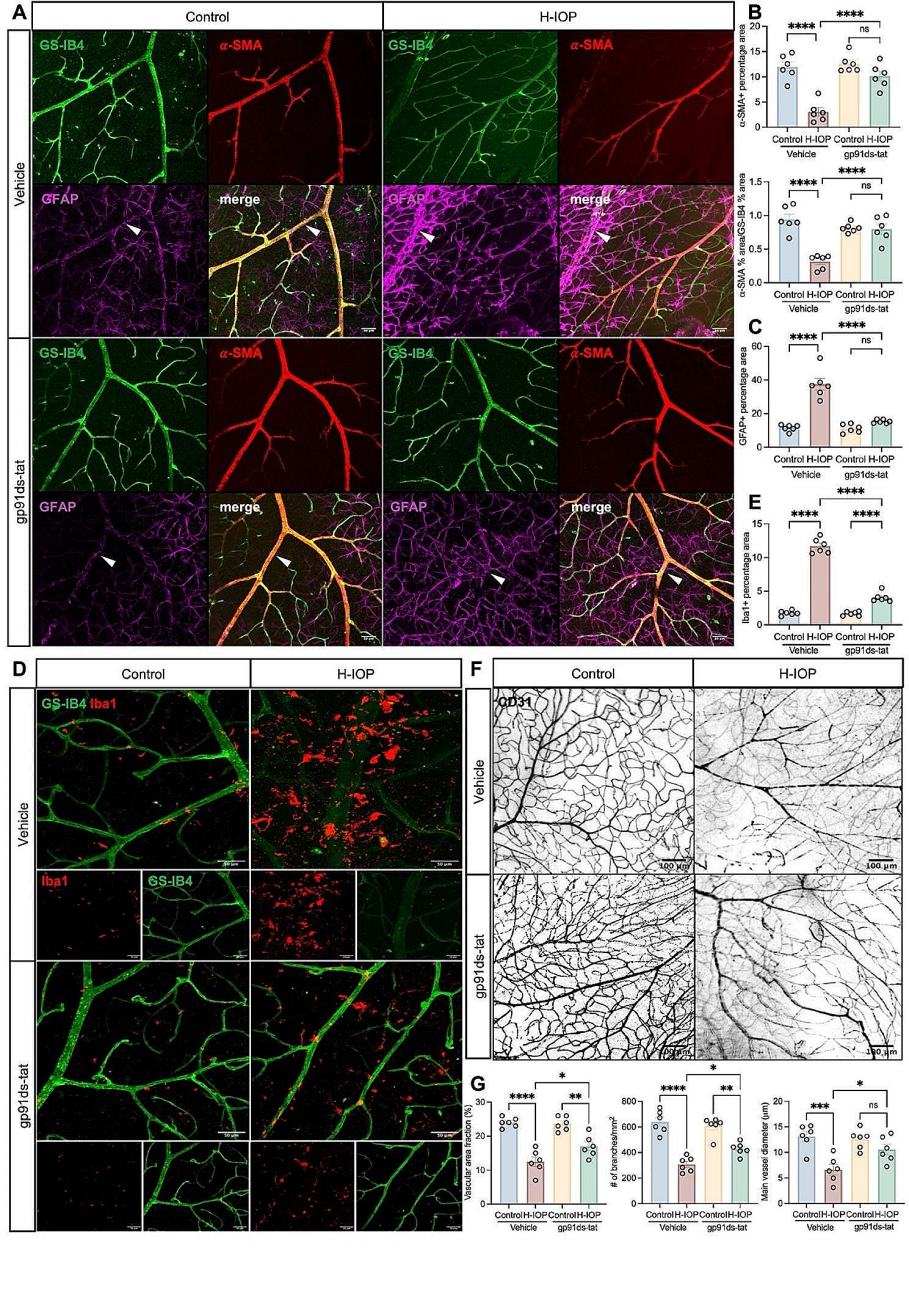
NOX2/ROS-activated glial cells involved in H-IOP-mediated blood-retinal barrier damage and inflammation. (**A**) Representative images of the iBRB stained by GS-IB4, α-SMA, and GFAP. Scale bar, 50 μm. (**B**) Analysis of the α-SMA positive % area and the α-SMA positive % area/GS-IB4 positive % area in the different groups (*n* = 6 in each group). (**C**) GFAP positive percentage area in the different groups (*n* = 6 in each group). (**D**) Representative immunostaining of Iba1 and GS-IB4 images of retinas after H-IOP treated by gp91ds-tat. Scale bar, 50 μm. (**E**) Analysis of Iba1 positive percentage area in the different groups (*n* = 6 in each group). (**F**) Representative immunostaining of CD31 images of retinas after H-IOP treated by gp91ds-tat. Scale bar, 100 μm. (**G**) Analysis of the vascular area fraction, the number of branches/mm^2^, and the main vessel diameter (µm) in the different groups (*n* = 6 in each group). Data are shown as mean ± SEM, one-way ANOVA with Tukey’s multiple comparisons test, *p < 0.05,**p < 0.01,***p < 0.001, ****p < 0.0001

Next, we hypothesized that the NOX2/ROS-specific expression pattern in the endothelium and vasculature might be involved in the regulation of the internal blood-retinal barrier (iBRB), a physiological barrier regulating the movement of ions, proteins, and water in and out of the retina, which characterizes ECs arrayed on the inner retinal microvascular system [44]. Therefore, we visualized the three main components in iBRB: glial cells (marked with GFAP, Iba1), ECs (labeled with GS-IB4), and pericytes (marked with α-SMA) in the retinal flat mount to explore further the contribution of NOX2 to iBRB (Fig. 6A). The results showed a significant decrease in the percentage coverage of α-SMA and the ratio of α-SMA/GS-IB4 after H-IOP (Fig. 6B), implicating damage to the retinal vascular system and vascular permeability. Besides, we employed confocal microscopy to observe the interaction of astrocytes with vascular units, which indicated astrocyte activation, with a significant increase in the percentage of GFAP-positive area, characterized by a significant increase in the coverage of their protrusions encircling the blood vessels (Fig. 6C). Consistent with our hypothesis, inhibition of the NOX2 activity effectively alleviated GFAP activation and proliferation adhering to the vascular surface after H-IOP, reversing the retinal vasculature system damage typified by a significant rebound in the ratio of α-SMA/GS-IB4 as evidenced by the significant rebound in the ratio of α-SMA/GS-IB4. Notably, we also observed significant activation (Amoeba-like shape) of Iba1 + microglia induced by H-IOP to converge and aggregate around the vasculature (Fig. 6D, E). In contrast, gp91ds-tat treatment significantly inhibited the activated state of microglia and interrupted the chemotaxis and aggregation of perivascular microglia (Fig. 6D, E). These findings suggest that the NOX2-dependent glial-endothelial unit plays a pivotal role in the structure and function of iBRB.

Meanwhile, we observed a dramatic decrease in capillary coverage with the tendency of the vasculature to simplify and degenerate in the retinas under H-IOP (Fig. 6F). Interestingly, this substantial remodeling could be dramatically ameliorated by gp91ds-tat, characterized by a 36% increase in vascular area fraction, 59% main vessel diameter, and 39% in branch number points (Fig. 6G).

**Fig. 7:**
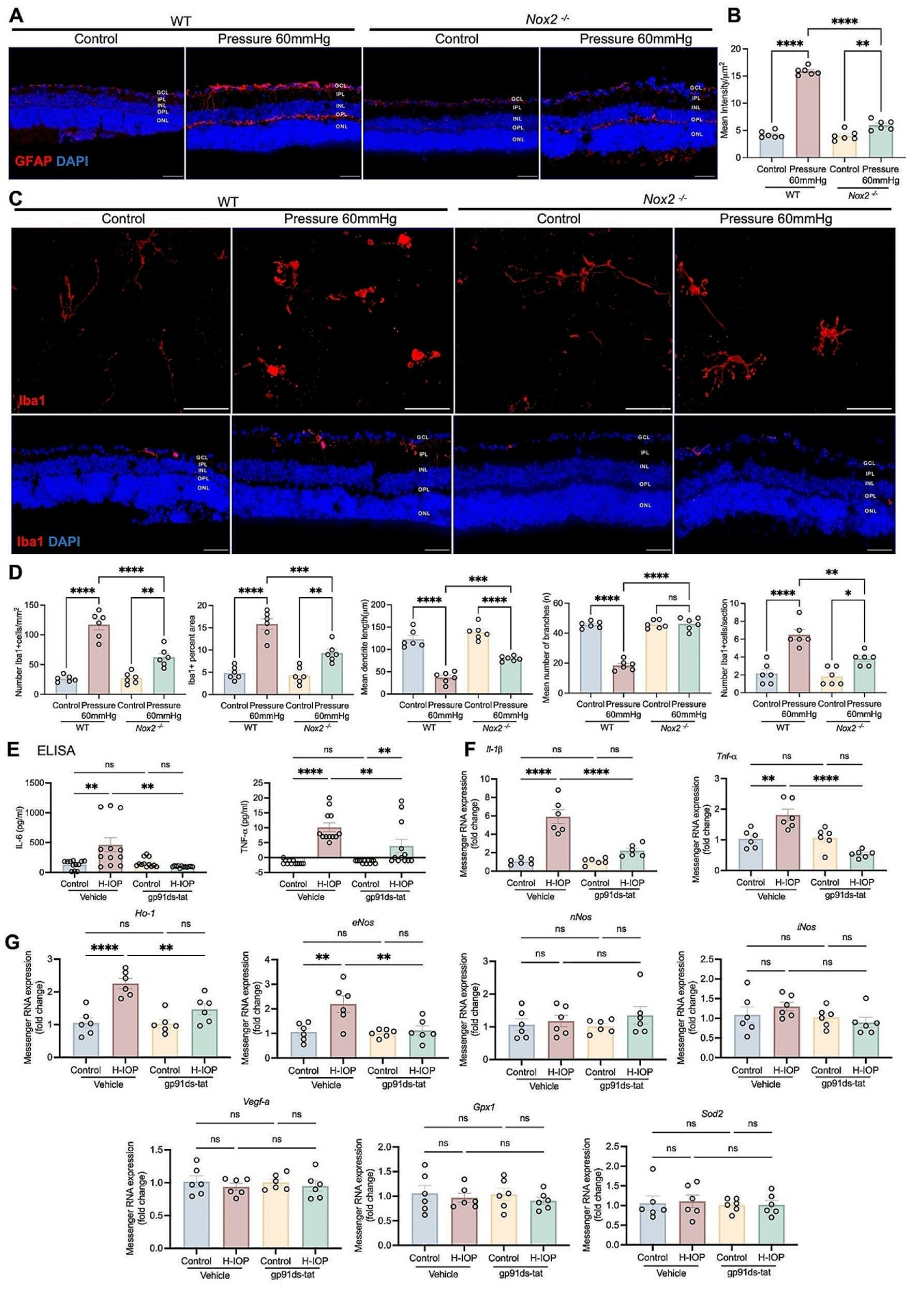
NOX2 deletion or pharmacological inhibition dampens pathologically high intraocular pressure injury-mediated reactive glial cell proliferation and neuroinflammation and identifies inflammatory and immune-related protein pathways and functional enrichment in experimental glaucoma. (**A**) Representative images of GFAP stained retinal cryosections from WT and Nox2^−/−^ mice after Pressure 60 mmHg 24 h. Scale bar, 50 μm. (**B**) Analysis of GFAP fluorescence mean intensity/µm^2^ (*n* = 6 in each group). (**C**) Representative images of Iba1 stained retinal flat-mounts and retinal cryosections from WT and Nox2^−/−^ mice after Pressure 60 mmHg 24 h. Scale bar, 50 μm. (**D**) Analysis of the number of Iba1 + cells/mm^2^, Iba1 + percent area, the mean dendrite length (µm), the mean number of branches (n), and the number of Iba1 positive cells per section (*n* = 6 in each group). (**E**) ELISA assay was performed to determine the retinal protein of TNF-α and IL-6 with gp91ds-tat treatment (*n* = 12 in each group). (**F**) Messenger RNA expression of genes coding for proinflammatory cytokines (Tnf-α and Il-1β) in different groups. Data are presented as the fold-change versus Veh-control (*n* = 6 in each group). (**G**) Messenger RNA expression of hypoxia genes (Vegf-a), antioxidant genes (Ho-1, Gpx1, Sod2), and nitric oxide synthases (eNos, iNos, and nNos) in different groups. Data are presented as the fold-change in retinas after H-IOP and with gp91ds-tat treatment versus control-vehicle (*n* = 6 in each group). Data are shown as mean ± SEM (one-way ANOVA with Tukey’s multiple comparisons test, *p < 0.05, **p < 0.01,***p < 0.001, ****p < 0.0001)

Mechanistically, NOX2/ROS is essential for the activation of glial cells, including Müller cells, astrocytes, and microglia, in which overactivation and gliosis are involved in vascular adhesion and pro-inflammatory responses, ultimately leading to the destruction of iBRB [45–47]. The retinas under Pressure 60 mmHg were observed to have an enormously increased and prolonged GFAP-positive process extending from the GCL to the INL, which effect was significantly inhibited in *Nox2* ^*−/−*^ retinas (Fig. 7A, B). Iba1 positive microglia are mainly migratory, increased in the GCL with an Amoeba-like shape, and a surge of amoeboid microglia with round bodies and scarce dendrites was detected after Pressure 60 mmHg. This microglia-reactive proliferation and infiltration could be dramatically alleviated by NOX2 deficiency (Fig. 7C, D).

Moreover, glial cell activation triggers an inflammatory response in the retinal vasculature by releasing pro-inflammatory mediators [48]. ELISA (Fig. 7E) and qPCR (Fig. 7F) results confirmed that inhibiting NOX2 activity could significantly reduce the H-IOP-induced surge in TNF-α, IL-1β, and IL-6 levels. In addition, we further examined the transcript levels of genes linked to angiogenesis and endothelial function by qPCR. The results indicated that H-IOP induces a significant elevation of *eNos* and *Ho-1*, which could be reversed by inhibiting NOX2 activity. In contrast, no significant differences in the mRNA levels of *Gpx1* and *Sod2*, hypoxia genes *Vegf-a*, *iNos*, and *nNos*, were found between the groups (Fig. 7G). Collectively, these data demonstrate that NOX2/ROS-activated glial cells facilitate iBRB damage and inflammation responses in glaucoma.

### NOX2/ET-1 axis mediates microglia activation switching to M1 pro-inflammatory phenotype via ERK1/2 pathway contributing to iBRB inflammatory injury

**Fig. 8:**
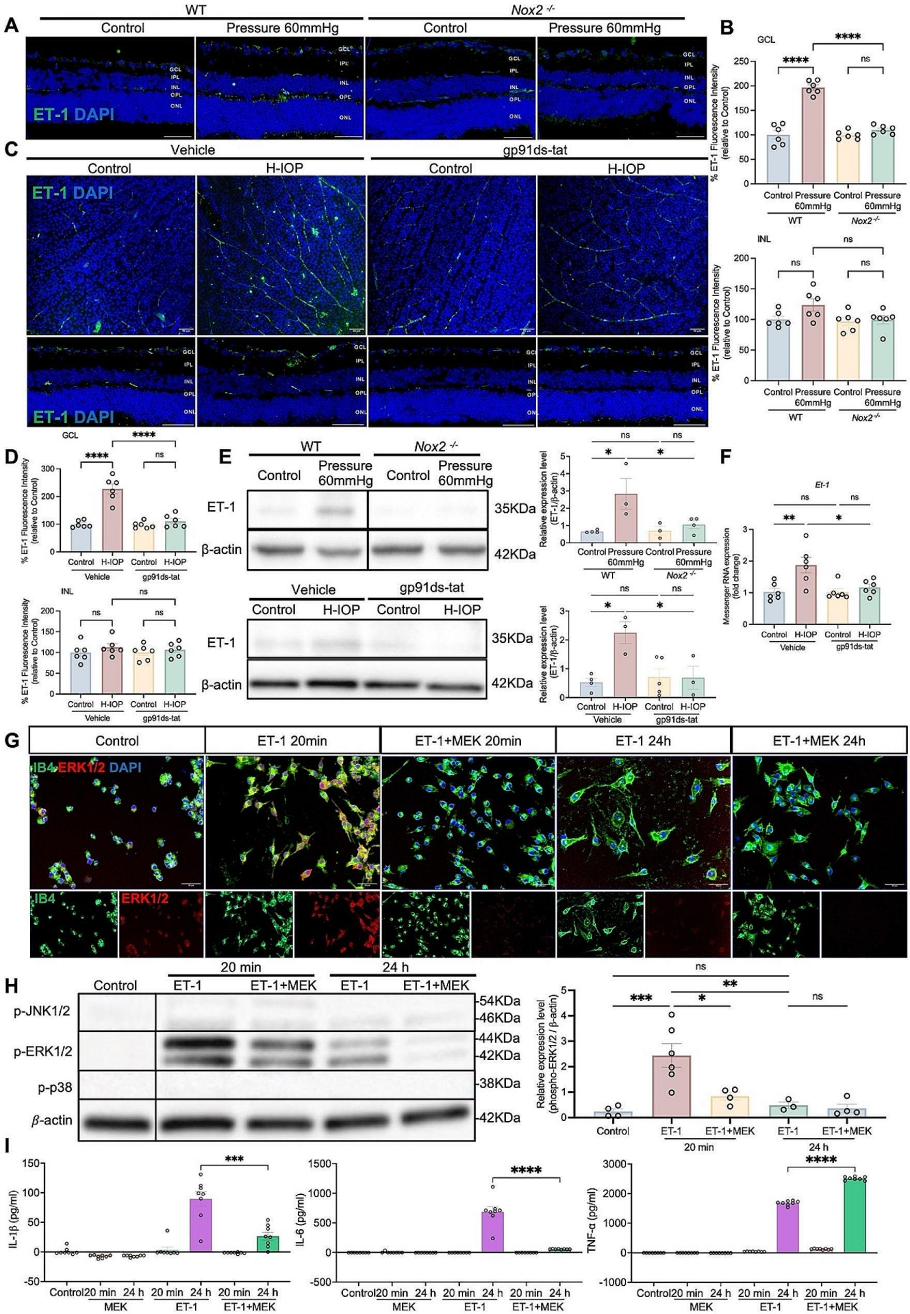
NOX2/ET-1 axis mediates microglia activation switching to M1 pro-inflammatory phenotype via ERK1/2 pathway contributing to iBRB inflammatory injury. (**A**) Representative images of ET-1 stained retinal cryosections from WT and Nox2^−/−^ after Pressure 60 mmHg 24 h. Scale bar, 50 μm. (**B**) Analysis of ET-1 fluorescence intensity in the GCL and INL. Data are presented as the percent fluorescence intensity of the groups versus control-WT (*n* = 6 in each group). (**C**) Representative images of ET-1 stained retinal flat-mount and cryosections after H-IOP with 500µM gp91ds-tat treatment. Scale bar, 50 μm. (**D**) Analysis of ET-1 fluorescence intensity in the GCL and INL. Data are presented as the percent fluorescence intensity of the groups versus Veh-control (*n* = 6 in each group). (**E**) Retinal extracts from WT and Nox2^−/−^ mice under Pressure 60 mmHg and H-IOP retinas with 500µM gp91ds-tat treatment were assayed for Western blot analysis of ET-1. Representative Western blots are presented. The results obtained from Western blot are expressed as the ratio to β-actin from 4–6 independent experiments. (**F**) Messenger RNA expression of Et-1 in retinas after H-IOP and with 500µM gp91ds-tat treatment. *n* = 6 in each group. (**G**) Representative images of IB4 and ERK1/2 stained microglia cultured by 100nM ET-1 and 20µM MEK 20–24 h. Scale bar, 50 μm. (**H**) Microglia lysis were assayed for Western blot analysis of p-JNK, p-ERK1/2 and p-p38 MAPK. Representative Western blots are presented. The results obtained from Western blot are expressed as the ratio to β-actin from 3 independent experiments. Data are shown as mean ± SEM, one-way ANOVA with Tukey’s multiple comparisons, *p < 0.05, **p < 0.01,***p < 0.001, ****p < 0.0001). (**I**) ELISA assay was performed to determine the cell protein of IL-6, IL-1β, and TNF-α (*n* = 8 in each group). Data are shown as mean ± SEM (unpaired t-test, ***p < 0.001, ****p < 0.0001)

Under pathophysiological conditions, intensified NOX2 activity promotes ET-1 production in ECs, which mediates pro-inflammatory microglia activation and neuroinflammation, involved in vascular disruption and disruption of the blood-brain barrier in neurodegenerative diseases [49–51]. To further explore the mechanism of NOX2 regulation of iBRB, we hypothesized that the NOX2/ET-1 axis regulates the proliferation of microglia (the principal mediator of neuroinflammation) via the MAPK signaling pathway mediating the inflammatory damage to the iBRB in pathologically H-IOP. We observed an effective attenuation of Pressure 60 mmHg-induced hyperimmunoreactivity of ET-1 in *Nox2* ^*−/−*^ retinas (Fig. 8A, B). Similarly, alterations in ET-1 immunoreactivity were verified in H-IOP retinas after our treatment with gp91ds-tat (Fig. 8C, D). Western blot analysis also demonstrated that Pressure 60 mmHg and H-IOP injury invokes a significant elevation in the protein level of ET-1 in retinal homogenates, which is reversible by genetic deletion of NOX2 and inhibition of gp91ds-tat (Fig. 8E). Also, gp91ds-tat significantly reversed the plunge in *Et-1* transcript levels caused by H-IOP (Fig. 8F).

To further elucidate the mechanism by which the NOX2/ET-1 axis mediates microglia activation, pure primary microglia cultures were first incubated with ET-1 (100 nM) for 20 min and 24 h. The results showed that ET-1 strongly promoted microglia activation after 24 h, while 20 min was not sufficient to activate microglia (Fig. 8G). Previous studies demonstrated the activation of intracellular pathways by ET-1, including PKC/ERK and p38MAPK [52]. To elucidate the molecular mechanism of ET-1 activation in microglia, we verified whether ET-1 could activate MAPK-dependent signaling pathways, including JNK, ERK1/2, and p38MAPK, in an experimental model. Interestingly, time-course analyses of microglial cultures confirmed that ET-1-induced phosphorylation of pERK1/2 isoforms appeared to drastically increase as early as 20 min after stimulation, while its expression level returned to physiological levels 24 h later (Fig. 8H). In contrast, the same stimulation pattern did not significantly affect the phosphorylation status of pJNK and p38 MAPK isoforms. Further, we treated the primary microglia with MEK1/2 (20µM) (a selective cell permeability inhibitor of the MEK/ERK pathway that blocks upstream kinase activation of MEK1 and MEK2) for 20 min and 24 h. The outcomes reveal that ET-1-induced pERK1/2 phosphorylation was remarkably blocked by MEK1/2 intervention at 20 min, whereas the phosphorylation levels of pJNK and p38 MAPK isoforms were not affected (Fig. 8H). Aiming to verify whether microglia activation is mediated by ERK1/2-dependent signaling, we visualized the co-localization of IB4 and ERK1/2 in microglia. As shown in Fig. 8G, relative to the untreated control group, microglial cell bodies exhibited a minor enlargement 20 min post-ET-1 application, accompanied by a marked increase in ERK1/2 expression, which showed co-localization with IB4. Following 24 h of ET-1 exposure, microglia assumed a characteristic activation phenotype, with ERK1/2 expression levels reverting to baseline. Moreover, we assayed markers of M1 (pro-inflammatory phenotype) to characterize ET-1-induced phenotypic shifts in the response of microglia. Figure 8I shows that ET-1 stimulation significantly increased the protein expression of IL-6, IL-1β, and TNF-α after 24 h of stimulation. Interestingly, with MEK1/2 intervention, the high expression of IL-6 and IL-1ß was significantly suppressed, while TNF-α was the only unaffected cytokine. These data suggest that ET-1 regulates the transition of microglia activation to an M1 pro-inflammatory phenotype through the ERK1/2 pathway.

## Discussion

**Fig. 9:**
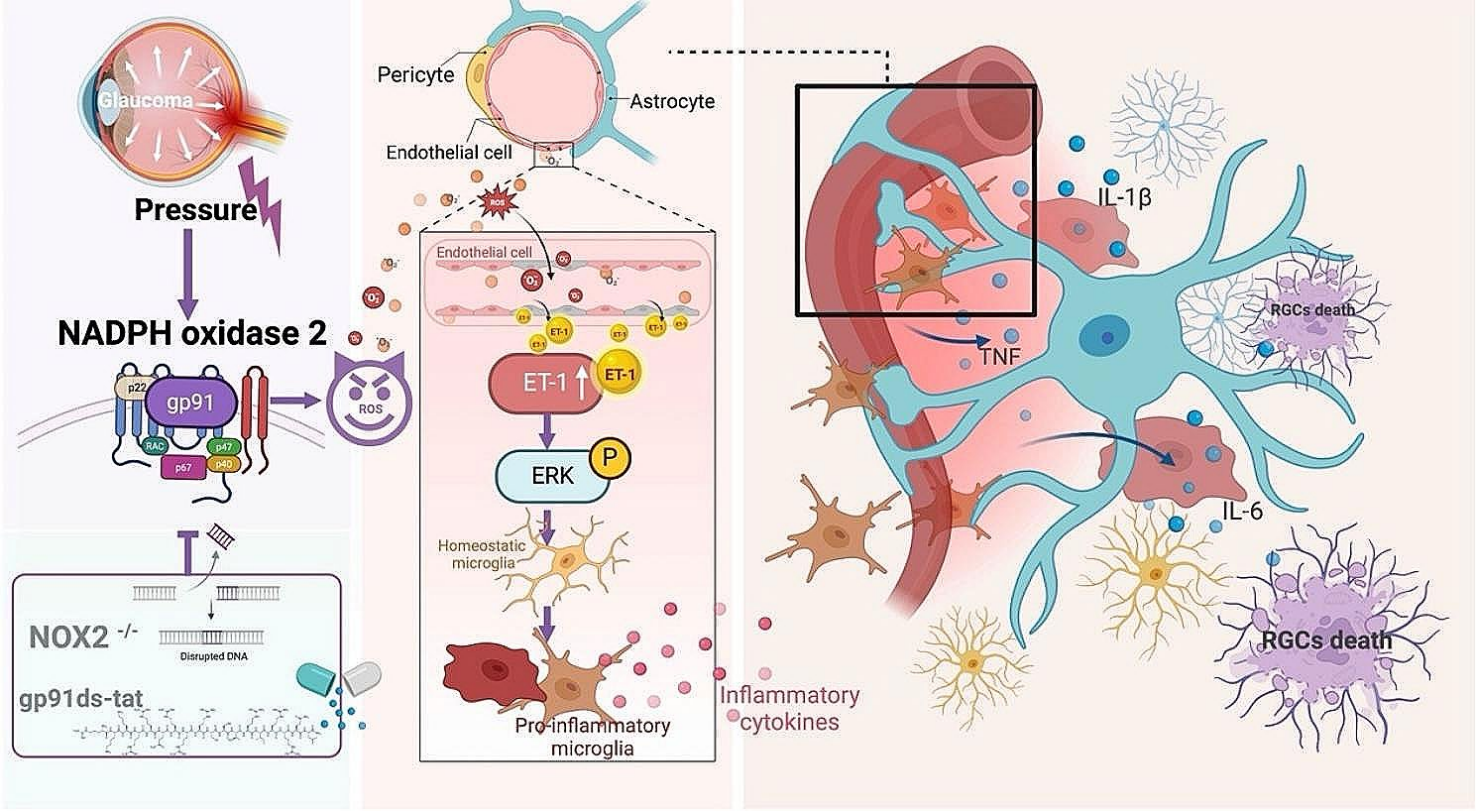
NADPH oxidase 2 deletion attenuates glaucomatous neurodegeneration induced by pathologically high intraocular pressure

In this study, we determined that NOX2 is the critical key to unlocking the “Pandora’s box” of oxidative stress under pathological H-IOP. We found that NOX2-mediated O_2_^-^ overproduction was mainly located in the vasculature and endothelium, which induced reactive glial cell proliferation that mediated neuroinflammation and iBRB injury. Mechanistically, NOX2/ROS induces endothelial-derived ET-1 overexpression, which activates the ERK1/2 signaling pathway to mediate the shift of microglia activation to a pro-inflammatory M1 phenotype that triggers the release of inflammatory mediators. However, NOX2-specific deletion or gp91ds-tat pharmacological inhibition of NOX2 activity effectively impairs ET-1 overexpression, which inhibits ERK1/2 transduction of microglia-mediated neuroinflammation, thereby rescuing glaucomatous RGC loss and ON axonal injury by attenuating iBRB injury and neurovascular unit (NVU) dysfunction (Fig. 9).

Recent evidence suggests that ON damage can continue despite effectively lowering IOP [53–55]. The retina is the most metabolically active tissue in the body [56]. Intraocular oxidative stress leads to direct RGC damage in glaucoma patients [57]. Post-optic nerve crush injury exhibited RGC death and increased mRNA expression of NOX1, NOX2, and NOX4 in the retina [58]. In the mouse retinal ischemia/reperfusion model, NOX2 and p22 ^phox^ gene expression were regulated [22]. NOX2 constitutes a crucial hub associated with oxidative stress in the retina after pathologically H-IOP. Previously, a study verified that NOX2 deficiency prevents apoptosis of RGC [22]. Although it is well-known that NOX2 is distributed throughout the retina [22], interestingly, NOX2 overexpression as a result of increased IOP predominantly takes place in the vasculature and GCL of the retina [19]. The vascular endothelium is responsible for regulating the passage of macromolecules and circulating cells from the blood to the tissues, which is a major target of oxidative stress [59]. In retinas with hyper-ophthalmia, mRNA levels of NOX2 are up-regulated, and the subsequent excessive ROS production contributes to reduced vascular endothelium-dependent diastole [19]. These findings address that NOX2 deficiency and gp91ds-tat confer neuroprotection by directly mitigating retinal oxidative stress, especially in ECs, vascular, and GCL, whereas it fails to influence IOP. Treatment with gp91ds-tat effectively reduces NOX2 mRNA expression, indicating decreased gene transcription [60]. Nevertheless, as shown by immunofluorescence, protein levels may not align with mRNA due to various post-transcriptional and post-translational factors. Despite NOX2 inhibitors reducing superoxide production [61, 62], NOX2’s complex regulation and other ROS-generating enzymes may result in ongoing or compensatory NOX2 activity, as detected by immunofluorescence. This implies that while NOX2 protein production might drop, active proteins could persist due to stability or post-transcriptional modifications, potentially explaining the increased immunofluorescence observed in Pressure 60 mmHg retinas treated by gp91ds-tat.

NOX2 promotes the development of inflammation, endothelial dysfunction, and ECs senescence in various vessels, including small retinal arteries [63–67]. Beyond ROS homeostasis, a complex cross-talk exists between individual endothelial-derived factors aimed at maintaining proper endothelial function. iBRB consists of retinal ECs, which are covered by astrocytes, pericytes, and end-footed Müller cells and are essential for maintaining the microenvironment of the inner retinal layers. In a rat model of non-arteritic anterior ischemic optic neuropathy with laser injury to the ON, NOX2 induction was again associated with microglia activation in the anterior ON [68]. In a unilateral H-IOP model evoked by cauterization, activation of astrocytes and microglia in the retina is accompanied by induction of NOX2 mRNA [69]. ROS production by NADPH oxidase can activate microglia to impair blood brain barrier (BBB) function [70]. BBB components are preserved and blood-brain barrier disruption is decreased when superoxide produced by NADPH oxidase is blocked with oleuropein [71]. Besides, the loss of pericytes, as well as the decreased pericyte-to-EC ratio, resulted in a localized increase in permeability, and the decreased pericyte coverage implied the central nervous system (CNS) microvascular dysfunction [72]. These data demonstrate that pathologically H-IOP stimulates the NOX2-dependent reactive proliferation of retinal glial cells and iBRB functional impairment. Dysregulation of this cross-talk leads to alterations in normal physiological processes in ECs, including vascular remodeling and impairment of endothelium-dependent vasorelaxation. In neurodegenerative diseases, microglia contract their protrusions and migrate toward the site of injury, where they release pro-inflammatory cytokines such as IL-1β, TNF-α, and IL-6 [73–75]. TNF-α could transmit ROS from neuronal cells to vascular cells [19] and also elicit NOX2-dependent ROS production in microglia [76] and is detected in the atrial fluid of glaucoma patients with TNF-α [77]. In our study, increased NADPH oxidase activity accompanied by upregulation of inflammatory cytokines (TNF-α, IL-6, and IL-1β) provides further support for the role of NADPH oxidase in vascular inflammation, consistent with previous studies [78]. Inhibition of NADPH oxidase activity alleviates H_2_O_2_-induced endothelial dysfunction via the NO/HO-1 pathway [79]. A previous study demonstrated that endothelial-specific overexpression of NOX2 was accompanied by increased levels of eNOS and SOD2 [80]. SOD2 upregulation is crucial for retinal oxidative balance, with its deficiency resulting in heightened oxidative stress markers and increased oxidative damage [81–83]. The absence of SOD2 upregulation under H-IOP conditions suggests a compromised retinal oxidative equilibrium and potential retinal damage risk.

Blockade of NOS greatly enhanced ET-1-induced endothelial contraction, and both endothelial removal of oleuropein and NADPH oxidase inhibition attenuated the enhanced contraction [84]. ET-1 concentrations were observed to be increased in separate animal models of glaucoma [85–87], and this peptide plays a role in hypertension-related vascular injury [88, 89]. NOX2 activity promotes ET-1 production in ECs [49], and NOX-mediated superoxide production contributes to ET-1-dependent regulation of vascular homeostasis in physiology and disease [90]. In cardiomyocytes, mechanical stretch-released Ang II [91] induces NOX2 activation of the auto-AT1 receptor and induces ET-1 release [92]. We clarified for the first time in retinal vessels, consistent with previous studies, that NOX2 deletion or inhibition of activity is followed by a corresponding alleviation of the H-IOP-induced elevated protein and transcript levels of ET-1, which mediates vascular oxidative stress, inflammation, and remodeling [93, 94]. As the perivascular area is covered by microglia and astrocytes [51], glial cells directly contact and encapsulate the vasculature, which directly affects the structure and function of the EC, rather than passive coexistence [95]. The direct role of ET-1 in reactive gliosis was demonstrated by in vivo infusion of exogenous ET or ET-R agonists, which leads to microglia and astrocyte proliferation [50, 94]. Furthermore, the effect of ET-1 on glial cell proliferation depends on c-Jun signaling through ERK- and JNK-dependent pathways [96]. The primary microglia were morphologically reactive and hypertrophied after the addition of ET-1 dependent on activation of the ERK1/2 pathway. Of particular note, ET-1 induced the activation of microglia toward the M1 pro-inflammatory phenotype, which is consistent with the previous findings [50]. Inhibition of the ERK1/2 pathway with PD98059, a MEK1/2-specific inhibitor, was sufficient to block IL-1β and IL-6 secretion by Listeria monocytogenes-infected macrophages completely and to reduce TNF-α secretion modestly [42]. Although MEK1/2 suppression could completely block IL-1β and IL-6 secreted by ET-1-induced pro-inflammatory phenotype of microglia M1, without affecting TNF-α secretion. The neuroglia that physically encapsulate the blood vessels are considered secondary barriers [97]. Through this close physical interaction between ECs and neuroglia, nutrients required for CNS function are transported from blood vessels to neurons primarily through neuroglia [98], while waste products are passed through neuroglia to microglia or back to the bloodstream [99]. Pro-inflammatory microglia cause damage by releasing inflammatory cytokines, amplifying inflammatory processes, and secondary neuronal death [100, 101]. The NVU glial cells are composed of neurons, vascular smooth muscle cells (VSMCs), pericytes, microglia, and vasculature [95]. NVU dysfunction is characterized by dysfunctional neurovascular coupling, neuronal death, neuroglial proliferation, microglia activation, mural cell migration, and BBB disruption [102, 103]. Typically, neuroglial dysfunction precedes neuronal and vascular lesions, which work closely with other NVU cells to sustain CNS function and maintenance [95].

## Conclusion

In summary, this study identifies NOX2 as a key driver of oxidative stress in the NVU under H-IOP, leading to glial proliferation, neuroinflammation, and iBRB damage. NOX2’s interaction with the ROS-ET-1-ERK1/2 axis fosters an inflammatory microglial response, exacerbating RGC and ON axonal injury. Counteracting NOX2 activity via genetic deletion or gp91ds-tat inhibitor not only dampens this inflammatory response but also preserves RGCs and ON integrity, highlighting a promising avenue for glaucoma therapy.

### Electronic supplementary material

Below is the link to the electronic supplementary material.

Supplementary Material 1

## Data Availability

No datasets were generated or analysed during the current study.

## Abbreviations

BBB: Blood brain barrier
BP: Biological processes
Brn3a: Brain-specific homeobox/POU domain protein 3A
CC: Cellular components
CNS: Central nervous system
CT: Comparative threshold
DCF: 2,7-dichlorofluorescein derivative 6-carboxy-2,7-dichlorodihydrofluorescein diacetate, di(acetoxymethyl ester)
DEPs: Differentially expressed proteins
DHE: Dye dihydroethidium
ECs: Endothelial cells
ELISA: Enzyme-linked immunosorbent assay
ET-1: Endothelin-1
FDR: False discovery rate
GCL: Ganglion cell layer
GFAP: Glial fibrillary acidic protein
GO: Gene Ontology
Gpx1: Glutathione peroxidases 1
H-IOP: High intra-ocular pressure
Ho-1: Heme oxygenase 1
iBRB: Internal blood-retinal barrier
IL-1β: Interleukin 1beta
IL-6: Interleukin 6
INL: Inner nuclear layer
IOP: Intraocular pressure
IPL: Inner plexiform layer
KEGG: Kyoto Encyclopedia of Genes and Genomes
LFQ: Label-free quantitation
MCE: Mixed Cellulose Ester
MF: Molecular function
NADPH: Nicotinamide adenine dinucleotide phosphate
NOS: Nitric oxide synthase
NOX: NADPH oxidase
NVU: Neurovascular unit
O_2_^-^: Superoxide
OCT: Optimal cutting temperature
ON: Optic nerve
ONL: Outer nuclear layer
OPL: Outer plexiform layer
PBS: Phosphate-buffered saline
PCA: Principal component analysis
PFA: Paraformaldehyde
PPD: p-phenylenediamine
Pressure 60 mmHg: Elevated hydrostatic pressure
RGC: Retinal ganglion cell
ROS: Reactive oxygen specie
SDS: Sodium dodecyl sulfate
Sod2: Superoxide dismutase type 2
Tbp: TATA-binding protein
TNF-α: Tumor necrosis factor-alpha
Vegf-a: Vascular endothelial growth factor-A
VSMCs: Vascular smooth muscle cells
WT: Wildtype
α-SMA: alpha smooth muscle actin

## References

[1] Tham Y-C (2014). Global prevalence of Glaucoma and projections of Glaucoma Burden through 2040: a systematic review and Meta-analysis. Ophthalmology.

[2] Weinreb RN, Khaw PT (2004). Primary open-angle glaucoma. Lancet.

[3] Shi X (2022). Oxidative stress, vascular endothelium, and the Pathology of Neurodegeneration in Retina. Antioxidants.

[4] Faraci FM. Reactive oxygen species: influence on cerebral vascular tone J Appl Physiol (1985). 2006;100(2):739 – 43. 10.1152/japplphysiol.01044.2005 16421281

[5] Himori N (2016). The association between systemic oxidative stress and ocular blood flow in patients with normal-tension glaucoma. Graefe’s Archive Clin Experimental Ophthalmol.

[6] Ju WK (2007). Elevated hydrostatic pressure triggers mitochondrial fission and decreases cellular ATP in differentiated RGC-5 cells. Invest Ophthalmol Vis Sci.

[7] Hernandez MR (2002). Differential gene expression in astrocytes from human normal and glaucomatous optic nerve head analyzed by cDNA microarray. Glia.

[8] Agapova OA, Kaufman PL, Hernandez MR (2006). Androgen receptor and NFkB expression in human normal and glaucomatous optic nerve head astrocytes in vitro and in experimental glaucoma. Exp Eye Res.

[9] Ruan Y, et al. Oxidative stress and vascular dysfunction in the retina: therapeutic strategies. Antioxidants (Basel); 2020;9:8. 10.3390/antiox9080761 PMC7465265 32824523

[10] Göllner M (2020). NOX2ko mice show largely increased expression of a mutated NOX2 mRNA encoding an inactive NOX2 protein. Antioxidants.

[11] Lambeth JD (2004). NOX enzymes and the biology of reactive oxygen. Nat Rev Immunol.

[12] Lassegue B, Clempus RE (2003). Vascular NAD (P) H oxidases: specific features, expression, and regulation. Am J Physiology-Regulatory Integr Comp Physiol.

[13] Fan Gaskin JC, Shah MH, Chan EC (2021). Oxidative stress and the role of NADPH oxidase in Glaucoma. Antioxidants.

[14] Henry E (1999). Peripheral endothelial dysfunction in normal pressure Glaucoma. Investig Ophthalmol Vis Sci.

[15] Sugiyama T (1995). Association of endothelin-1 with normal tension glaucoma: clinical and fundamental studies. Surv Ophthalmol.

[16] Kaiser HJ (1995). Endothelin-1 plasma levels in normal-tension glaucoma: abnormal response to postural changes. Graefe’s Archive Clin Experimental Ophthalmol.

[17] Noske W, Hensen J, Wiederholt M (1997). Endothelin-like immunoreactivity in aqueous humor of patients with primary open-angle glaucoma and cataract. Graefe’s Archive Clin Experimental Ophthalmol.

[18] Wilkinson-Berka JL (2009). Identification of a retinal aldosterone system and the protective effects of mineralocorticoid receptor antagonism on retinal vascular pathology. Circul Res.

[19] Gericke A et al. Elevated intraocular pressure causes abnormal reactivity of mouse retinal arterioles Oxidative medicine and cellular longevity, 2019. 2019. 10.1155/2019/9736047 PMC6954472 31976030

[20] Bode K (2023). Unlocking the power of NOX2: a comprehensive review on its role in immune regulation. Redox Biol.

[21] Urner S (2020). NADPH oxidase inhibition: preclinical and clinical studies in diabetic complications. Antioxid Redox Signal.

[22] Yokota H (2011). Neuroprotection from retinal ischemia/reperfusion injury by NOX2 NADPH oxidase deletion. Investig Ophthalmol Vis Sci.

[23] Ruiz-Ederra J, Verkman AS (2006). Mouse model of sustained elevation in intraocular pressure produced by episcleral vein occlusion. Exp Eye Res.

[24] Chen H (2015). Progressive Degeneration of Retinal and Superior Collicular Functions in mice with sustained ocular hypertension. Investig Ophthalmol Vis Sci.

[25] Thomson BR, et al. Angiopoietin-1 knockout mice as a genetic model of Open-Angle Glaucoma. Translational Vision Science & Technology; 2020;9(4):16–16. 10.1167/tvst.9.4.16 PMC7396191 32818103

[26] Cai H, Griendling KK, Harrison DG (2003). The vascular NAD (P) H oxidases as therapeutic targets in cardiovascular diseases. Trends Pharmacol Sci.

[27] Rey F (2001). Novel competitive inhibitor of NAD (P) H oxidase assembly attenuates vascular O2 – and systolic blood pressure in mice. Circul Res.

[28] Fawell S (1994). Tat-mediated delivery of heterologous proteins into cells. Proc Natl Acad Sci.

[29] Lian H, Roy E, Zheng H. Protocol for primary Microglial Culture Preparation. Bio Protoc, 2016. 6(21). 10.21769/BioProtoc.1989 PMC5669279 29104890

[30] Miller WP (2018). Deletion of the Akt/mTORC1 Repressor REDD1 prevents visual dysfunction in a Rodent Model of type 1 diabetes. Diabetes.

[31] Zadeh JK et al. Apolipoprotein e deficiency causes endothelial dysfunction in the mouse retina Oxidative medicine and cellular longevity, 2019. 2019. 10.1155/2019/5181429 PMC6875001 31781340

[32] Wang M (2022). Intraocular pressure-Induced endothelial dysfunction of retinal blood vessels is persistent, but does not trigger retinal ganglion cell loss. Antioxidants.

[33] Oelze M (2006). NADPH oxidase accounts for enhanced superoxide production and impaired endothelium-dependent smooth muscle relaxation in BKβ1–/– mice. Arterioscler Thromb Vasc Biol.

[34] Stein JD, Khawaja AP, Weizer JS (2021). Glaucoma in Adults-Screening, diagnosis, and management: a review. JAMA.

[35] Tonner H (2022). A monoclonal Anti-HMGB1 antibody attenuates neurodegeneration in an experimental animal model of Glaucoma. Int J Mol Sci.

[36] Chidlow G (2010). Spatiotemporal characterization of optic nerve degeneration after chronic hypoperfusion in the rat. Investig Ophthalmol Vis Sci.

[37] Wisniewski J (2009). Universal sample preparation method for proteome analysis. Nat Meth.

[38] Karpievitch YV, Dabney AR, Smith RD (2012). Normalization and missing value imputation for label-free LC-MS analysis. BMC Bioinformatics.

[39] Laspas P (2019). The M1 muscarinic acetylcholine receptor subtype is important for retinal neuron survival in aging mice. Sci Rep.

[40] Ruan Y (2020). Oxidative stress and vascular dysfunction in the retina: therapeutic strategies. Antioxidants.

[41] Chan EC (2009). Regulation of cell proliferation by NADPH oxidase-mediated signaling: potential roles in tissue repair, regenerative medicine and tissue engineering. Pharmacol Ther.

[42] Herb M (2019). Mitochondrial reactive oxygen species enable proinflammatory signaling through disulfide linkage of NEMO. Sci Signal.

[43] Fan Gaskin JC, Shah MH, Chan EC. Oxidative stress and the role of NADPH oxidase in Glaucoma. Antioxid (Basel), 2021. 10(2). 10.3390/antiox10020238 PMC7914994 33557289

[44] O’Leary F, Campbell M (2023). The blood-retina barrier in health and disease. Febs j.

[45] Bringmann A (2006). Müller cells in the healthy and diseased retina. Prog Retin Eye Res.

[46] Ravelli KG (2019). Nox2-dependent neuroinflammation in an EAE Model of multiple sclerosis. Transl Neurosci.

[47] Surace MJ, Block ML (2012). Targeting microglia-mediated neurotoxicity: the potential of NOX2 inhibitors. Cell Mol Life Sci.

[48] Jha MK, Jeon S, Suk K (2012). Glia as a link between Neuroinflammation and Neuropathic Pain. Immune Netw.

[49] Kamhieh-Milz J (2023). Ang II promotes ET-1 production by regulating NOX2 activity through transcription factor Oct-1. Arterioscler Thromb Vasc Biol.

[50] Abdul Y (2020). Endothelin-1 (ET-1) promotes a proinflammatory microglia phenotype in diabetic conditions. Can J Physiol Pharmacol.

[51] Zlokovic BV (2008). The blood-brain barrier in health and chronic neurodegenerative disorders. Neuron.

[52] Schinelli S (2001). Stimulation of endothelin B receptors in astrocytes induces cAMP response element-binding protein phosphorylation and c-fos expression via multiple mitogen-activated protein kinase signaling pathways. J Neurosci.

[53] Group CN-TGS (1998). Comparison of glaucomatous progression between untreated patients with normal-tension glaucoma and patients with therapeutically reduced intraocular pressures. Am J Ophthalmol.

[54] Lichter PR (2001). Interim clinical outcomes in the collaborative initial Glaucoma treatment study comparing initial treatment randomized to medications or surgery. Ophthalmology.

[55] Heijl A (2002). Reduction of intraocular pressure and glaucoma progression: results from the early manifest Glaucoma trial. Arch Ophthalmol.

[56] Tokarz P, Kaarniranta K, Blasiak J (2013). Role of antioxidant enzymes and small molecular weight antioxidants in the pathogenesis of age-related macular degeneration (AMD). Biogerontology.

[57] Tezel G (2006). Oxidative stress in glaucomatous neurodegeneration: mechanisms and consequences. Prog Retin Eye Res.

[58] Yamamoto K (2014). The novel rho kinase (ROCK) inhibitor K-115: a new candidate drug for neuroprotective treatment in glaucoma. Investig Ophthalmol Vis Sci.

[59] Hadi HA, Carr CS, Al J, Suwaidi (2005). Endothelial dysfunction: cardiovascular risk factors, therapy, and outcome. Vasc Health Risk Manag.

[60] Singh PK (2022). Specific inhibition of NADPH oxidase 2 modifies chronic epilepsy. Redox Biol.

[61] Maqbool A (2020). Divergent effects of genetic and pharmacological inhibition of Nox2 NADPH oxidase on insulin resistance-related vascular damage. Am J Physiol Cell Physiol.

[62] Sukumar P (2013). Nox2 NADPH oxidase has a critical role in insulin resistance-related endothelial cell dysfunction. Diabetes.

[63] Zadeh JK (2019). Responses of retinal arterioles and ciliary arteries in pigs with acute respiratory distress syndrome (ARDS). Exp Eye Res.

[64] Rojas M (2017). NOX2-induced activation of arginase and diabetes-induced retinal endothelial cell senescence. Antioxidants.

[65] Fan LM (2017). Aging-associated metabolic disorder induces Nox2 activation and oxidative damage of endothelial function. Free Radic Biol Med.

[66] Cahill-Smith S, Li JM (2014). Oxidative stress, redox signalling and endothelial dysfunction in ageing‐related neurodegenerative diseases: a role of NADPH oxidase 2. Br J Clin Pharmacol.

[67] Konior A (2014). NADPH oxidases in vascular pathology. Antioxid Redox Signal.

[68] Mehrabian Z (2017). Oligodendrocyte death, neuroinflammation, and the effects of minocycline in a rodent model of nonarteritic anterior ischemic optic neuropathy (rNAION). Mol Vis.

[69] Sapienza A (2016). Bilateral neuroinflammatory processes in visual pathways induced by unilateral ocular hypertension in the rat. J Neuroinflamm.

[70] Sumi N (2010). Lipopolysaccharide-activated microglia induce dysfunction of the blood–brain barrier in rat microvascular endothelial cells co-cultured with microglia. Cell Mol Neurobiol.

[71] Yenari MA (2006). Microglia potentiate damage to blood–brain barrier constituents: improvement by minocycline in vivo and in vitro. Stroke.

[72] Bonkowski D (2011). The CNS microvascular pericyte: pericyte-astrocyte crosstalk in the regulation of tissue survival. Fluids Barriers CNS.

[73] Kreutzberg GW (1996). Microglia: a sensor for pathological events in the CNS. Trends Neurosci.

[74] Clayton DF, George JM (1999). Synucleins in synaptic plasticity and neurodegenerative disorders. J Neurosci Res.

[75] Becher B, Prat A, Antel JP (2000). Brain-immune connection: immuno‐regulatory properties of CNS‐resident cells. Glia.

[76] Geng L (2020). Nox2 dependent redox-regulation of microglial response to amyloid-β stimulation and microgliosis in aging. Sci Rep.

[77] Soto I, Howell GR. The complex role of neuroinflammation in glaucoma. Cold Spring Harb Perspect Med, 2014. 4(8). 10.1101/cshperspect.a017269 PMC4109578 24993677

[78] Zhang L (2003). Diabetes-induced oxidative stress and low-grade inflammation in porcine coronary arteries. Circulation.

[79] Luo M, Tian R, Lu N (2019). Nitric oxide protected against NADPH oxidase-derived superoxide generation in vascular endothelium: critical role for heme oxygenase-1. Int J Biol Macromol.

[80] Bendall JK (2007). Endothelial Nox2 overexpression potentiates vascular oxidative stress and hemodynamic response to angiotensin II: studies in endothelial-targeted Nox2 transgenic mice. Circul Res.

[81] Sandbach JM (2001). Ocular pathology in mitochondrial superoxide dismutase (Sod2)-deficient mice. Invest Ophthalmol Vis Sci.

[82] Usui S (2011). Overexpression of SOD in retina: need for increase in H2O2-detoxifying enzyme in same cellular compartment. Free Radic Biol Med.

[83] Justilien V (2007). SOD2 knockdown mouse model of early AMD. Invest Ophthalmol Vis Sci.

[84] Sánchez A (2014). Endothelin-1 contributes to endothelial dysfunction and enhanced vasoconstriction through augmented superoxide production in penile arteries from insulin-resistant obese rats: role of ET(A) and ET(B) receptors. Br J Pharmacol.

[85] Källberg ME (2002). Endothelin 1 levels in the aqueous humor of dogs with glaucoma. J Glaucoma.

[86] Thanos S, Naskar R (2004). Correlation between retinal ganglion cell death and chronically developing inherited glaucoma in a new rat mutant. Exp Eye Res.

[87] Prasanna G (2005). Effect of elevated intraocular pressure on endothelin-1 in a rat model of glaucoma. Pharmacol Res.

[88] Maki S (2004). The endothelin receptor antagonist ameliorates the hypertensive phenotypes of transgenic hypertensive mice with renin-angiotensin genes and discloses roles of organ specific activation of endothelin system in transgenic mice. Life Sci.

[89] Rautureau Y (2015). Inducible human endothelin-1 overexpression in endothelium raises blood pressure via endothelin type A receptors. Hypertension.

[90] Meyer MR, Barton M, Prossnitz ER (2014). Functional heterogeneity of NADPH oxidase-mediated contractions to endothelin with vascular aging. Life Sci.

[91] Sadoshima J-i (1993). Autocrine release of angiotensin II mediates stretch-induced hypertrophy of cardiac myocytes in vitro. Cell.

[92] Ito H (1993). Endothelin-1 is an autocrine/paracrine factor in the mechanism of angiotensin II-induced hypertrophy in cultured rat cardiomyocytes. J Clin Investig.

[93] Amiri F (2004). Endothelium-restricted overexpression of human endothelin-1 causes vascular remodeling and endothelial dysfunction. Circulation.

[94] Amiri F (2008). Vascular inflammation in absence of blood pressure elevation in transgenic murine model overexpressing endothelin-1 in endothelial cells. J Hypertens.

[95] Kugler EC, Greenwood J, MacDonald RB. The Neuro-glial-vascular unit: the role of Glia in neurovascular unit formation and dysfunction. Front Cell Dev Biology, 2021. 9. 10.3389/fcell.2021.732820 PMC8502923 34646826

[96] Gadea A, Schinelli S, Gallo V (2008). Endothelin-1 regulates astrocyte proliferation and reactive gliosis via a JNK/c-Jun signaling pathway. J Neurosci.

[97] Kutuzov N, Flyvbjerg H, Lauritzen M (2018). Contributions of the glycocalyx, endothelium, and extravascular compartment to the blood–brain barrier. Proc Natl Acad Sci.

[98] Hurley JB, Lindsay KJ, Du J (2015). Glucose, lactate, and shuttling of metabolites in vertebrate retinas. J Neurosci Res.

[99] Marina N (2018). Brain metabolic sensing and metabolic signaling at the level of an astrocyte. Glia.

[100] Brown GC, Neher JJ (2014). Microglial phagocytosis of live neurons. Nat Rev Neurosci.

[101] Hu X (2015). Microglial and macrophage polarization—new prospects for brain repair. Nat Reviews Neurol.

[102] Zlokovic BV (2005). Neurovascular mechanisms of Alzheimer’s neurodegeneration. Trends Neurosci.

[103] Willis CL (2011). Glia-induced reversible disruption of blood–brain barrier integrity and neuropathological response of the neurovascular unit. Toxicol Pathol.

