# Supplementary material for: Pathological high intraocular pressure induces glial cell reactive proliferation contributing to neuroinflammation of the blood-retinal barrier via the NOX2/ET-1 axis-controlled ERK1/2 pathway
Source: J Neuroinflammation. 2024 Apr 22;21:105. doi: 10.1186/s12974-024-03075-x (PMC11034147; doi:10.1186/s12974-024-03075-x)
Supplement: Supplementary file 1 — Supplementary Material 1 [file 12974_2024_3075_MOESM1_ESM.pptx]

## Slide 1
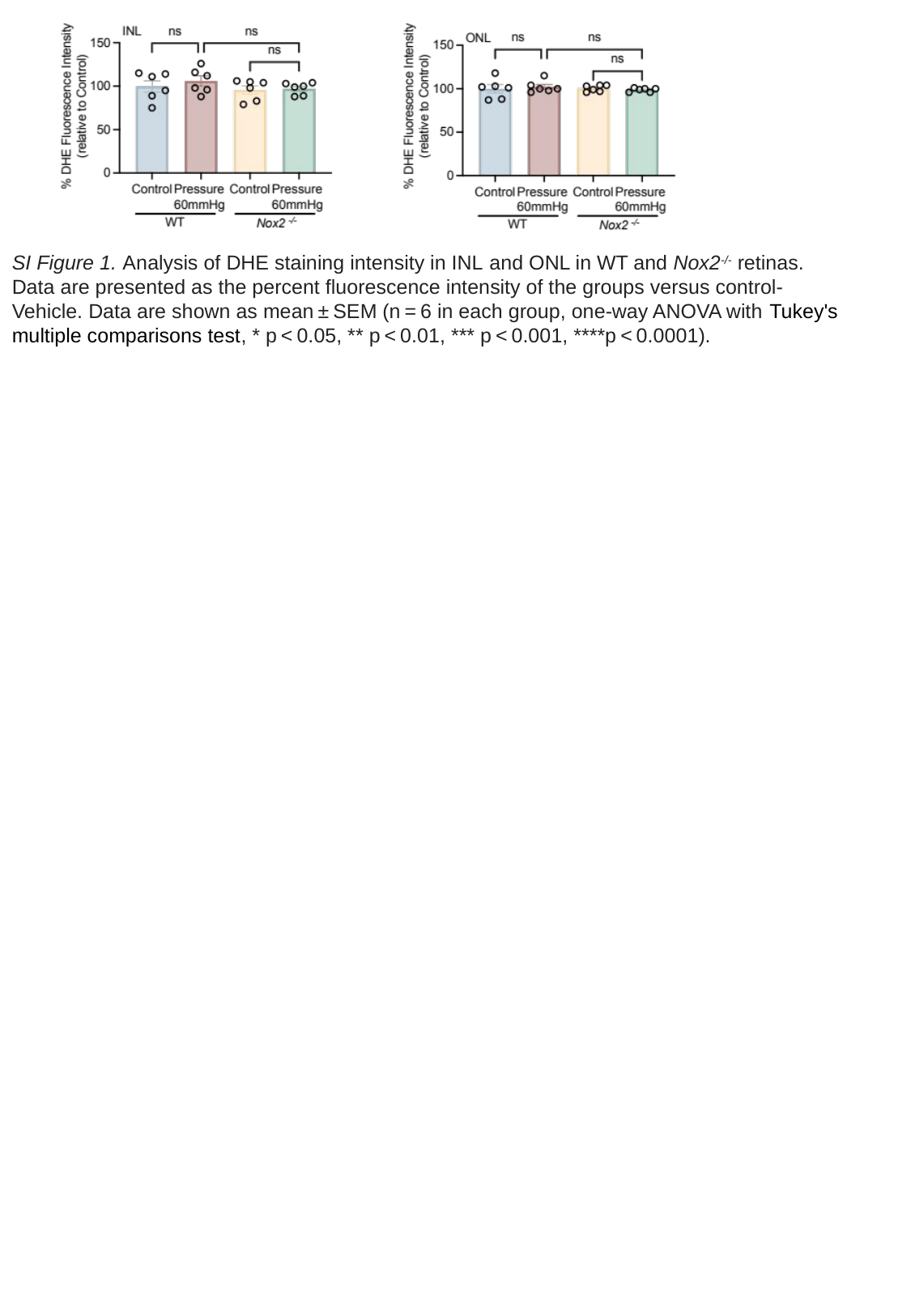

SI Figure 1. Analysis of DHE staining intensity in INL and ONL in WT and Nox2-/- retinas. Data are presented as the percent fluorescence intensity of the groups versus control-Vehicle. Data are shown as mean ± SEM (n = 6 in each group, one-way ANOVA with Tukey's multiple comparisons test, * p < 0.05, ** p < 0.01, *** p < 0.001, ****p < 0.0001).
